# Influence of drought stress on the metabolite and ion composition in nectar and nectaries of different day‐ and night‐flowering *Nicotiana* species

**DOI:** 10.1111/plb.70000

**Published:** 2025-02-18

**Authors:** T. Göttlinger, D. Naegel, J. E. Dick, G. Lohaus

**Affiliations:** ^1^ Molecular Plant Science/Plant Biochemistry University of Wuppertal Wuppertal Germany

**Keywords:** Amino acid composition, drought stress, floral nectar, ion composition, sugar composition

## Abstract

- The frequencies of droughts worldwide will increase in the future due to climate changes. Nectar composition of plant species varies in relation to pollinator and can also be influenced by drought. We investigated both different pollinated species and the effects of drought in parallel. In addition, the influence of drought on nectar production and metabolism in nectaries should be investigated, since very little is currently known about this.
- The influence of drought stress on nectaries, nectar and leaves of 4 day‐ and night‐flowering *Nicotiana* species (pollinated by sunbirds, hummingbirds, hawkmoths or bats) were investigated. The nectar volume, as well as metabolite concentrations (sugars, amino acids), inorganic ions and starch were measured. PCA and PERMANOVA were applied to determine the relative importance of different drought conditions on metabolism of nectaries and nectar.
- Drought stress led to changes in composition of nectaries and nectar in all four *Nicotiana* species. The day‐flowering species had relatively similar changes, whereas the night‐flowering species differed from these and also from each other. Quantities of sugars, amino acids and inorganic ions per flower decreased sharply in all *Nicotiana* species because of a strong decrease in nectar volume.
- Drought stress not only compromises plant growth but also nectar secretion and composition. These changes are likely to affect plant–pollinator interactions and may negatively impact successful pollination.

## Graphical abstract

Drought stress leads to reduced nectar secretion and alters nectar composition of day‐ and night‐flowering *Nicotiana* species, which may impair plant–pollinator interactions.

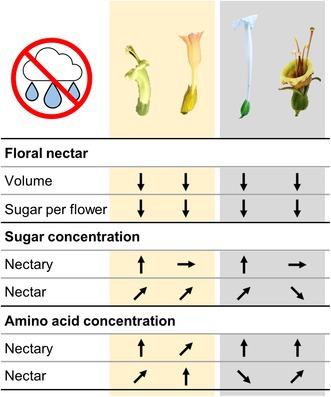

## INTRODUCTION

The global climate is changing rapidly and extreme climate events, such as droughts, are predicted to increase in frequency, duration and severity, which will have serious impacts on agriculture and plant growth (Dai 2013; Dietz *et al*. 2021; Gautam *et al*. 2023). Limited water availability represents a major constraint on growth, development and production of crops (Ciais *et al*. 2005). In addition, drought stress affects the reproductive properties of plants, with consequences for pollinators and successful plant reproduction (Borghi *et al*. 2019).

Flowering plants produce nectar as reward for animal pollinators that cross‐pollinate flowers when they collect nectar (Baker & Baker 1983; Kessler *et al*. 2012; Nicolson 2022). Nectar is a sugar‐rich watery liquid that is produced and secreted by special glands called nectaries (Fahn 1979). The main sugars in nectar are the hexoses glucose and fructose and the disaccharide sucrose. In addition, nectar contains, to a lesser extent, amino acids, inorganic ions, and other secondary compounds (Baker & Baker 1973, 1983; Seo *et al*. 2013; Nicolson 2022). The nectar produced by flowering plants can vary in quantity and composition, depending on the plant species (Baker & Baker 1973, 1983; Kessler *et al*. 2012; Göttlinger *et al*. 2019). Furthermore, composition of floral nectar, especially the ratio of sucrose‐to‐hexoses, has often been related to the pollinator type of different plant species (Baker & Baker 1983; Tiedge & Lohaus 2017; Göttlinger *et al*. 2019). Nectar composition can also be influenced by environmental factors, such as heat or water availability, as such conditions alter the metabolism of the entire plant, including the flowers (Halpern *et al*. 2010; Borghi *et al*. 2019; Rering *et al*. 2020).

The influence of drought stress on plant–pollinator interaction as well as on flower, pollen and nectar traits has been investigated in various species, especially in bee‐pollinated plants (Carroll *et al*. 2001; Brown *et al*. 2016; Descamps *et al*. 2021a). This influence manifests as a reduction in number of flowers and reduced nectar volume (Waser & Price 2016; Göttlinger & Lohaus 2020; Rering *et al*. 2020; Kuppler *et al*. 2021; Descamps *et al*. 2021a). The influence of drought on total sugar concentration in nectar is unclear. Some studies describe a decrease in concentration of sugars in nectar (Descamps *et al*. 2018), while others found no changes or increased sugar concentration (Carroll *et al*. 2001). Such changes in floral traits can, in turn, have a negative impact on the attraction of pollinators and thus on plant reproductive success. Therefore, drought stress will directly affect plant reproductive success by altering plant physiology, but also indirectly through effects on pollinators (Rering *et al*. 2020).

However, studies on drought stress in plants with different pollinator types or parallel analyses of nectaries and nectar are rare (Göttlinger & Lohaus 2020). In particular, the influence of drought stress on metabolism in nectaries has hardly been investigated. Furthermore, it is not yet well understood how drought stress affects nectar compounds other than sugars, such as amino acids or inorganic ions (Descamps *et al*. 2021a). The genus *Nicotiana* (Solanaceae) is ideally suited for such analyses because members of this genus differ in flower morphology, flowering time (including day‐ and night‐flowering species) and main pollinators (including sunbirds, hummingbirds, moths and bats). Floral nectar of *Nicotiana* species is produced in nectaries located at the base of the gynoecium (Carter *et al*. 1999).

The objective of this study was to analyse the influence of drought stress on different compounds in nectar of *Nicotiana* species having various pollinators. In addition, the nectaries were also analysed to gain insight into biochemical causes of changes in nectar and regulation of nectar composition. Previous studies have shown that, depending on the plant species and pollinator type, sugar composition in nectar varies. While the nectar of *N. africana* (pollinated by sunbirds) and *N. otophora* (pollinated by bats) is rich in hexoses, the nectar of *N. tabacum* (pollinated by hummingbirds) and *N. sylvestris* (pollinated by hawkmoths) is rich in sucrose (Kaczorowski *et al*. 2005; Tiedge & Lohaus 2017, 2018). Moreover, the concentrations of amino acids and inorganic ions in nectar also varies in these species (Tiedge & Lohaus 2017).

Such results raise the question as to whether drought stress leads to changes in nectar composition in the above four *Nicotiana* species. Therefore, nectar and nectaries of control and drought‐stressed *Nicotiana* plants were analysed in parallel for sugars, amino acids and inorganic ion content. To compare biochemical changes in nectaries with changes in whole plants, leaves of the plants were also analysed.

## MATERIAL AND METHODS

### Plant material and growth conditions

**Fig. 1:**
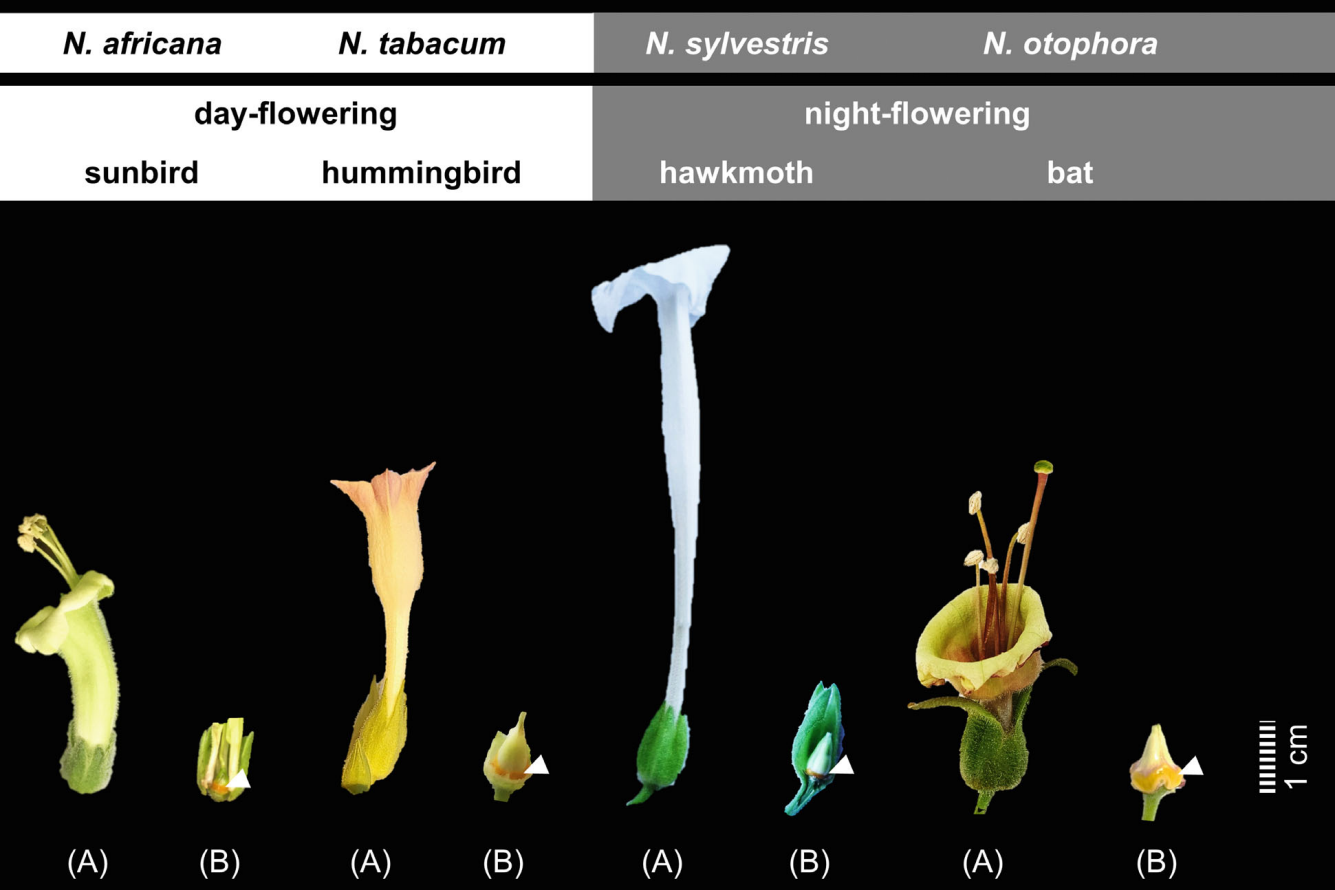
Flowers and nectaries of the analysed *Nicotiana* species. The *Nicotiana* species include 2 day‐flowering and two night‐flowering species. The day‐flowering species are *N. africana* (Nectariniidae; pollinated by sunbirds) and *N. tabacum* (Trochilidae; pollinated by hummingbirds). The night‐flowering species are *N. sylvestris* (Sphingidae; pollinated by hawkmoths) and *N. otophora* (Glossophaginae; pollinated by bats). For each species, a picture of the flower (A) and exposed nectaries (B) are provided. In (B), nectaries are marked with a white arrow.

Seeds of *N. africana* (Merxm.), *N. tabacum* (L.) (‘Badischer Burley E’), *N. sylvestris* (Speg. & Comes) and *N. otophora* (Griseb.) were obtained from NiCoTa (Rheinstetten, Germany). The *Nicotiana* species used in this study can be divided into day‐ and night‐flowering species (Fig. 1). The day‐pollinated species are *N. africana*, pollinated mainly by sunbirds (Marlin *et al*. 2014), and *N. tabacum*, pollinated by hummingbirds (Tiedge & Lohaus 2017). The night‐flowering species are *N. sylvestris*, pollinated by hawkmoths (Chase *et al*. 2010), and *N. otophora*, pollinated by nectar‐feeding bats (Nattero *et al*. 2003). Plants of each species were individually potted in 5 L pots containing compost and grown in a closed greenhouse at the University of Wuppertal (Germany). The plants were cultivated under a 14‐h light/10‐h dark cycle, irradiance of ca. 300 μmol photons m^−2^ s^−1^ and 25°C day/18°C night.

Plants with the first fully grown flowers on the four *Nicotiana* species were exposed to drought induced by not watering the pots. At the same time, control plants of all *Nicotiana* species received sufficient water (soil humidity ca. 50%). In each run, three plants of each species were placed under drought and three were used as controls. At the beginning of the trial, samples were collected from all plants of the respective species. After 7–8 days without watering, further samples were collected from the same plants (mild drought stress; soil humidity 15–20%), and from control plants (well‐watered) for comparison. After 14–17 days without watering, samples were taken again (severe drought stress; soil humidity <10%), and also from control plants (well‐watered) for comparison. At each time‐period, samples of leaves, nectaries and nectar were collected. At the beginning of experiments, control and experimental plants were similar in appearance and metabolite concentrations in leaves, nectaries or nectar. Furthermore, control plants (well‐watered) showed no differences at the time of mild and severe drought stress and were therefore grouped as ‘control’ for each *Nicotiana* species.

### Collection of leaves, nectaries and nectar

For each tissue (leaves, nectaries) at least four samples, and for nectar at least eight samples of each *Nicotiana* species were collected from four plants. All samples were harvested 3 to 4 h after anthesis, then immediately frozen in liquid nitrogen and stored at −80°C until further analysis. For leaf material, samples (~200 mg) were taken with a razor blade (Göttlinger *et al*. 2019). Each sample (~100 mg) of nectary tissue comprised 5–10 nectaries, depending on species. A scalpel was used to remove nectary tissue from the base of the ovary in the flower (Fig. 1). This tissue was then washed with ultrapure water to remove any external sugars (Göttlinger & Lohaus 2022). After anthesis, a nectar sample was collected from a single flower using a micropipette, and each nectar sample was analysed separately. The volume of nectar from flowers of the different *Nicotiana* species varied between 10 and 200 μl. Furthermore, no microbial contamination of nectar samples from any species could be detected microscopically.

### Water content of leaf and nectary tissue

To analyse the water content of leaf and nectary tissue, samples of each tissue from each *Nicotiana* species were weighed, dried, then reweighed. The ratio of dry to fresh weight represents the water content of the leaf or nectary tissue (Tiedge & Lohaus 2018).

### Extraction of soluble metabolites from leaf and nectary tissue

Soluble metabolites (sugars, amino acids) and inorganic ions were extracted from nectary or leaf tissue using chloroform:methanol:water extraction (Nadwodnik & Lohaus 2008). For this, 200 mg milled leaf material and 100 mg milled nectary material frozen in liquid nitrogen were used.

### Analysis of metabolites (sugars, free amino acids) and inorganic ions in leaves, nectaries and nectar

Nectar samples, extracts from nectaries and from leaf tissue were analysed using HPLC to determine concentration sand composition of sugars, amino acids and inorganic ions. Different sugars in the collected material were isocratically detected via an ICS‐5000 HPIC system (Thermo Fisher Scientific) using an anion exchange column and pulse amperometric detector for data collection (Lohaus & Schwerdtfeger 2014). An Ultimate 3000 HPLC system (Thermo Fisher Scientific) with a reversed‐phase column (Merck LiChroCART^®^ 125‐4 using Superspher^®^ 100 RP‐18 endcapped) was used for detection of free amino acids (alanine, arginine, aspartate, asparagine, glutamate, glutamine, glycine, histidine, isoleucine, leucine, lysine, methionine, phenylalanine, proline, serine, threonine, tryptophan, tyrosine, valine) in each plant material using a fluorescence detector (Göttlinger *et al*. 2019).

Inorganic anions (chloride, phosphate, sulphate) and cations (potassium, sodium, magnesium, calcium) were analysed separately via HPLC with an anion or cation exchange column for separation and a conductivity detector for analysis (Lohaus *et al*. 2001). Chromatograms were evaluated with an integration program using a calibration curve for each component (Chromeleon 7.2).

### Calculation of metabolite and inorganic ion concentrations in leaves and nectaries

By measuring metabolite (sugar, amino acids) or ion content in leaves and nectaries as μmol g^−1^ fresh weight (FW) and water content of leaves and nectaries, it was also possible to determine metabolite or ion concentration (mM) in both tissues (Tiedge & Lohaus 2018; Göttlinger *et al*. 2019).

### Analyses of starch in leaves and nectaries

The insoluble residue of the chloroform:methanol:water extract from leaf and nectary samples were treated with KOH, α‐amylase and amyloglucosidase to cleave starch into glucose. Aliquots (50 μl) of each incubation mixture were analysed spectrophotometrically for glucose. The starch content was calculated as milligrams of glucose equivalents per gram fresh weight (Lunn & Hatch 1995).

### Statistical analysis

All statistical analyses were performed with R software (v. 4.4.0, www.r‐project.org). The significance of differences between metabolite, inorganic ion and starch concentrations was determined in more than two groups using one‐way ANOVA, followed by Tukey *post‐hoc* tests. When comparing two groups to assess significance, a *t*‐test was used.

For Principal Components Analysis (PCA) data from each tissue (leaf, nectary, nectar) and each *Nicotiana* species (*N. africana*, *N. tabacum*, *N. sylvestris*, *N. otophora*) were used separately (Göttlinger *et al*. 2019). For the drought stress treatment, this was divided into groups: control, mild drought stress and severe drought stress. For a balance within the PCA, the groups were from equal sample numbers. In addition, PERMANOVA was applied to determine the relative importance of different drought treatments on the metabolites and inorganic ions in leaf, nectary tissue and nectar (Anderson 2014; Göttlinger *et al*. 2019). In addition, Permutational Analysis of Multivariate Dispersions (PERMDISP) was performed to test the extent to which significance in PERMANOVA is caused by location and dispersion effects (Anderson 2006).

## RESULTS

The influence of drought stress on leaves, nectaries and nectar was studied in 2 day‐flowering and two night‐flowering *Nicotiana* species (Fig. 1). The day‐flowering *N. africana* is pollinated by sunbirds (Nectariniidae) and *N. tabacum* by hummingbirds (Trochilidae), while the night‐flowering *N. sylvestris* is pollinated by hawkmoths (Sphingidae) and *N. otophora* by nectar‐feeding bats (Glossophaginae). The effects of drought stress to plants were visible, among other things, as reduced water content of leaves and plants with signs of wilting (Table S1).

### Influence of drought treatment on flower opening and nectar volume

**Fig. 2:**
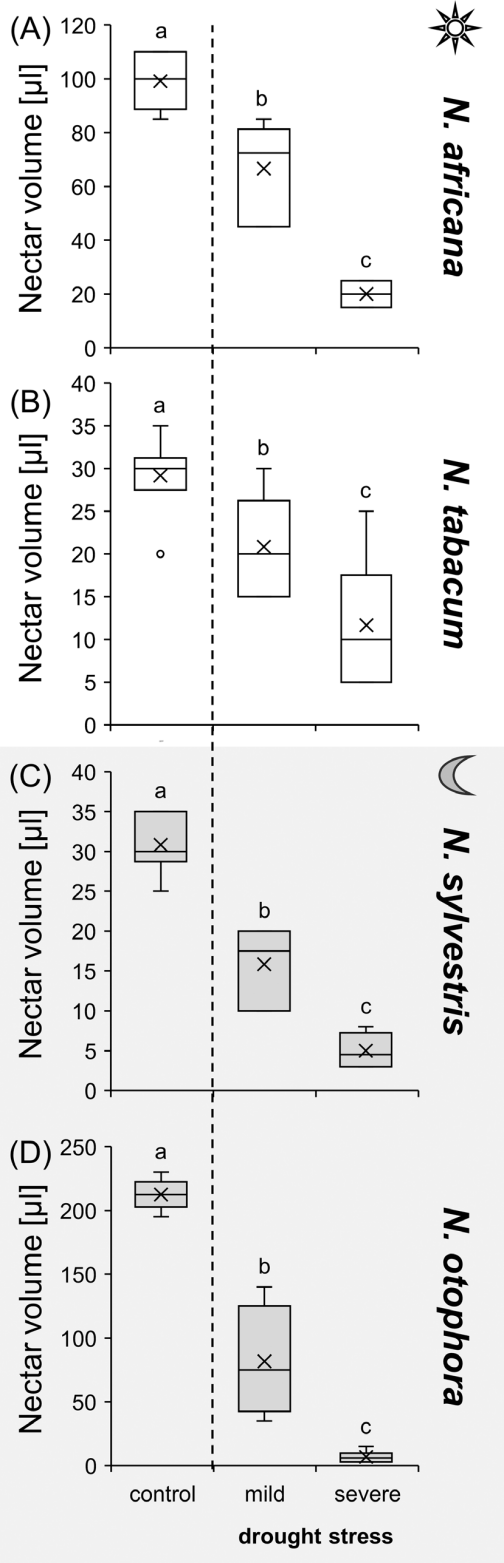
Nectar volume of four *Nicotiana* species under different drought treatments (control, mild, severe). The *Nicotiana* species include 2 day‐pollinated and two night‐pollinated species. The day‐pollinated species are *N. africana* (A) and *N. tabacum* (B). The night‐pollinated species are *N. sylvestris* (C) and *N. otophora* (D). Different letters represent significant differences in nectar volume between treatments with drought (Tukey HSD; *P* < 0.05; *n* = 6).

Under control conditions, all four *Nicotiana* species produced a large number of flowers per plant, with five to nine flowers opening each day, depending on species (Table S2). Under drought stress, flower formation and number of flowers opening per day decreased to two to three flowers until finally no flowers opened. In addition, under drought, nectar volume decreased by at least 60% in day‐flowering species (*N. tabacum* 60%, *N. africana* 80%) and by at least 80% in night flowering species (*N. sylvestris* 80%, *N. otophora* 90%) (Fig. 2).

### Sugar concentrations in leaves, nectaries and nectar under drought stress conditions

**Fig. 3:**
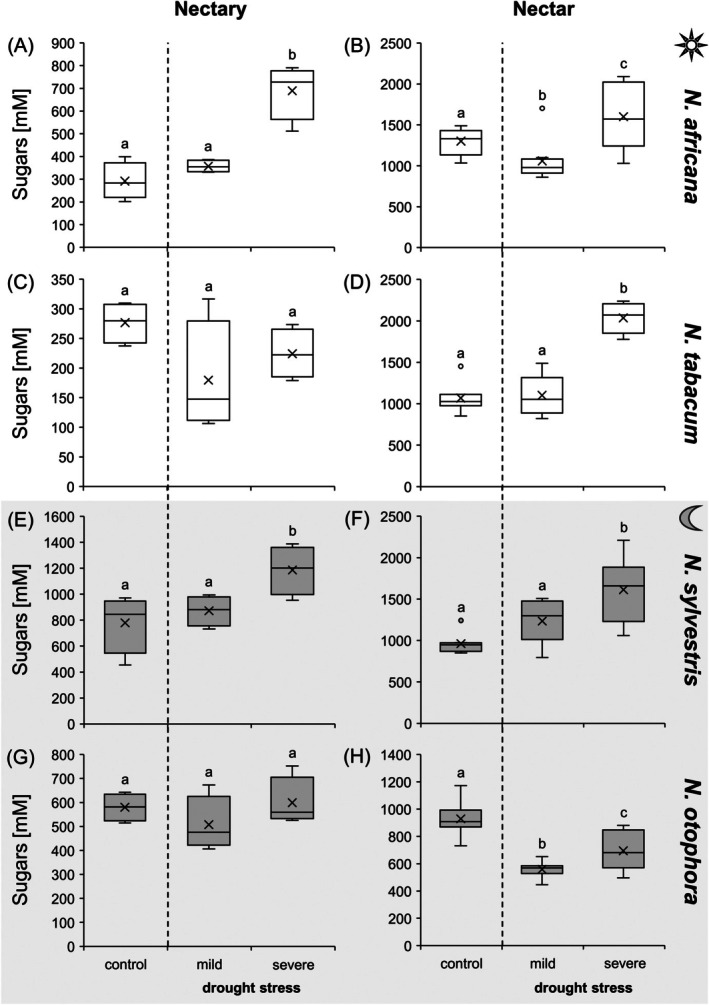
Sum of sugars (glucose, fructose, sucrose) in nectaries and nectar of four *Nicotiana* species under different drought treatments (control, mild, severe). To directly compare sugar concentrations in nectaries (A, C, E, G) and nectar (B, D, F, H), both are presented with the same unit. *Nicotiana* species include 2 day‐flowering (A–D) and two night‐flowering (E–H) species. The day‐flowering species are *N. africana* (A & B) and *N. tabacum* (C & D). The night‐flowering species are *N. sylvestris* (E & F) and *N. otophora* (G & H). Different letters represent significant differences in nectar volume between treatments with drought (Tukey HSD; *P* < 0.05; nectaries *n* = 4; nectar: *n* = 8).

The leaves, nectaries and nectar of the *Nicotiana* species contained high amounts of sugars with different proportions of glucose, fructose, and sucrose. No other sugars were detected in significant amounts (Tables S3–S6). Among the four *Nicotiana* species, under control conditions, total sugar concentration in nectaries was higher in the night‐flowering species (600–800 mM) than in the day‐flowering species (~300 mM; Fig. 3). Mild drought stress did not affect sugar concentrations in nectaries, as these were similar under control (well‐watered) and mild drought stress (Fig. 3). In *N. tabacum* and *N. otophora*, sugar concentrations did not change significantly even under severe drought stress, whereas in *N. africana* and *N. sylvestris* there was a significant increase in total sugar concentration (Fig. 3A,E). A significant increase in total sugar concentration was also observed in leaves of *N. africana* and *N. sylvestris* under severe drought stress (Fig. S1). Sugar concentration in the nectar was 900–1,100 mM, only *N. africana* had a higher sugar concentration (1,400 mM). In three species (*N. africana*, *N. tabacum*, *N. sylvestris*) there was a significant increase in total sugar concentrations in nectar, up to 2000 mM, during drought stress (Fig. 3B,D,F). In contrast, a slight decrease in total sugar concentration (from 900 to 700 mM) was observed in the bat‐pollinated species, *N. otophora* (Fig. 3H).

**Fig. 4:**
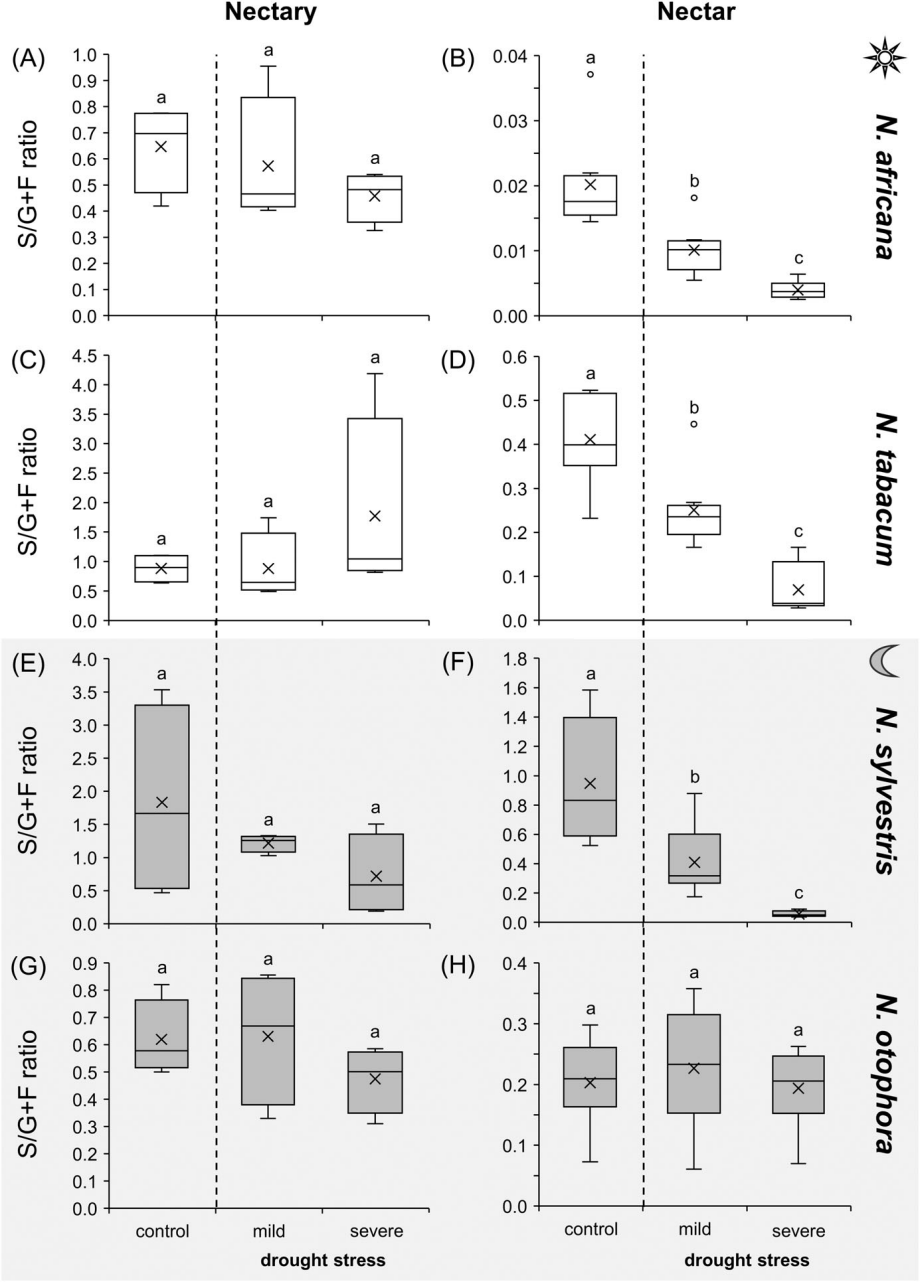
Sucrose‐to‐hexoses ratio (ref. mM) in nectaries and nectar of four *Nicotiana* species under different drought treatments (control, mild, severe). *Nicotiana* species include 2 day‐flowering (A–D) and two night‐flowering (−H) species. The day‐flowering species are *N. africana* (A & B) and *N. tabacum* (C & D). The night‐flowering species are *N. sylvestris* (E & F) and *N. otophora* (G & H). Different letters represent significant differences in nectar volume, respectively, between the treatments with drought (Tukey HSD; *P* < 0.05; nectaries *n* = 4; nectar: *n* = 8).

The sucrose‐to‐hexose ratio in nectaries was 0.6 (*N. otophora*) to 1.6 (*N. sylvestris*; Fig. 4, data from Tables S3–S6). There were no significant changes in these sugar ratios during drought stress (Fig. 4A,C,E,G). The same applied to the sucrose‐to‐hexose ratios in leaves, which did not change even under drought stress (Fig. S2). In general, the nectar of all species had a lower sucrose‐to‐hexose ratio than the nectaries (Fig. 4). In the 2 day‐flowering species and the night‐flowering species *N. sylvestris*, the sucrose‐to‐hexose ratio in nectar decreased significantly, which means that the concentration of hexoses increased whereas the concentration of sucrose decreased and the ratio approached zero (Fig. 4B,D,F). In contrast, *N. otophora* showed no significant changes and the sucrose‐to‐hexose ratio was ca. 0.2 over the entire drought stress period (Fig. 4H).

### Amino acid concentrations in nectaries and nectar under drought stress

**Fig. 5:**
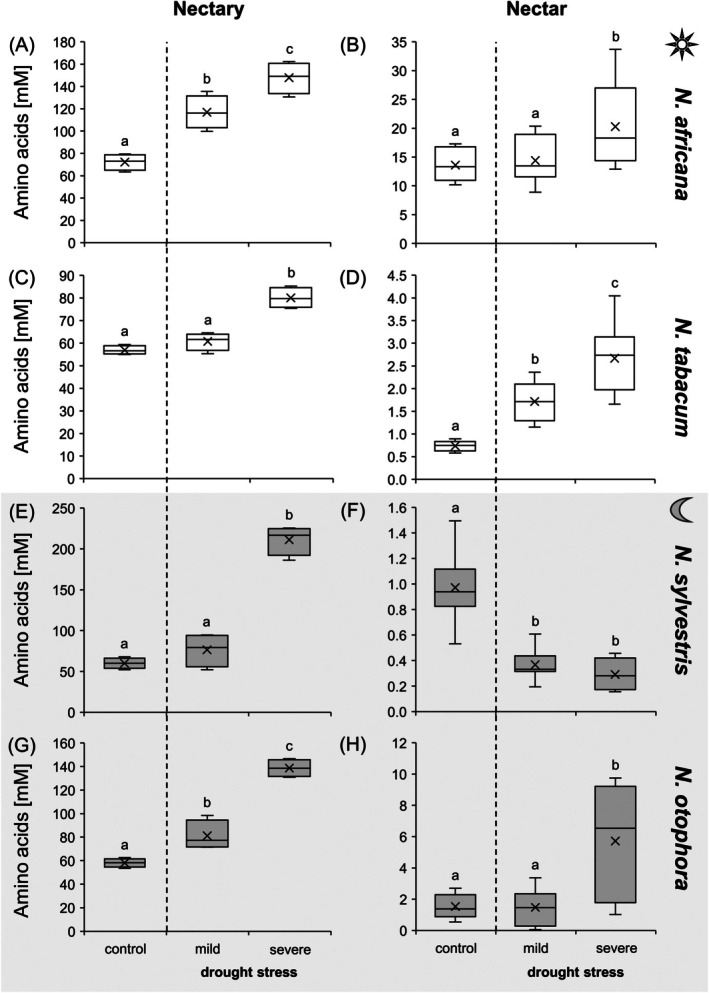
Sum of amino acids (Ala, Arg, Asp, Asn, Gln, Glu, Gly, His, Ile, Leu, Lys, Met, Phe, Pro, Ser, Thr, Trp, Tyr, Val) in nectaries and nectar of four *Nicotiana* species under different drought treatments (control, mild, severe). To directly compare amino acid concentrations in nectaries (A, C, E, G) and nectar (B, D, F, H), both are presented with the same unit. The *Nicotiana* species include 2 day‐flowering (A–D) and two night‐flowering species (E–H). The day‐flowering species are *N. africana* (A & B) and *N. tabacum* (C & D). The night‐flowering species are *N. sylvestris* (E & F) and *N. otophora* (G & H). Different letters represent significant differences in nectar volume between treatments with drought (Tukey HSD; *P* < 0.05; nectaries *n* = 4; nectar: *n* = 8).

Total amino acid concentrations were lower than the sugar concentrations in nectaries and in nectar. In the nectaries of all four *Nicotiana* species, total amino acid concentration was ca. 60 mM under control conditions (Fig. 5, Tables S3–S6). Under both mild and subsequent severe drought stress to plants, total amino acid concentration initially increased slightly and later significantly (Fig. 5A,C,E,G). In *N. sylvestris*, the concentration increased from 60 to 220 mM in nectaries (Fig. 5E), in *N. africana* and *N. otophora* to 140–150 mM (Fig. 5A,G), while in *N. tabacum* the increase was only to 80 mM (Fig. 5C). Under drought stress, amino acid concentration in leaves also increased, at least in *N. africana* and *N. otophora* (Fig. S3). Compared to nectaries, nectar always contained significantly lower concentrations of amino acids. The total amino acid concentration in nectar was highest in *N. africana* at 13 mM (Fig. 5B) and <2 mM in the other *Nicotiana* species (Fig. 5D,F,H). Under severe drought stress the amino acid concentration increased in *N. africana*, *N. tabacum* and *N. otophora* (Fig. 5B,D,H). In contrast, *N. sylvestris* showed a significant decrease in amino acid concentration, from 0.9 to 0.3 mM, even under mild drought stress (Fig. 5F).

It should be noted that the increase in amino acid concentrations in leaves, nectaries and nectar of *Nicotiana* species under drought stress was caused by an increase in amino acids (Table S7). However, the change in concentration of individual amino acids in the different species and in the different tissues did not show any consistent trend (Table S7).

**Fig. 6:**
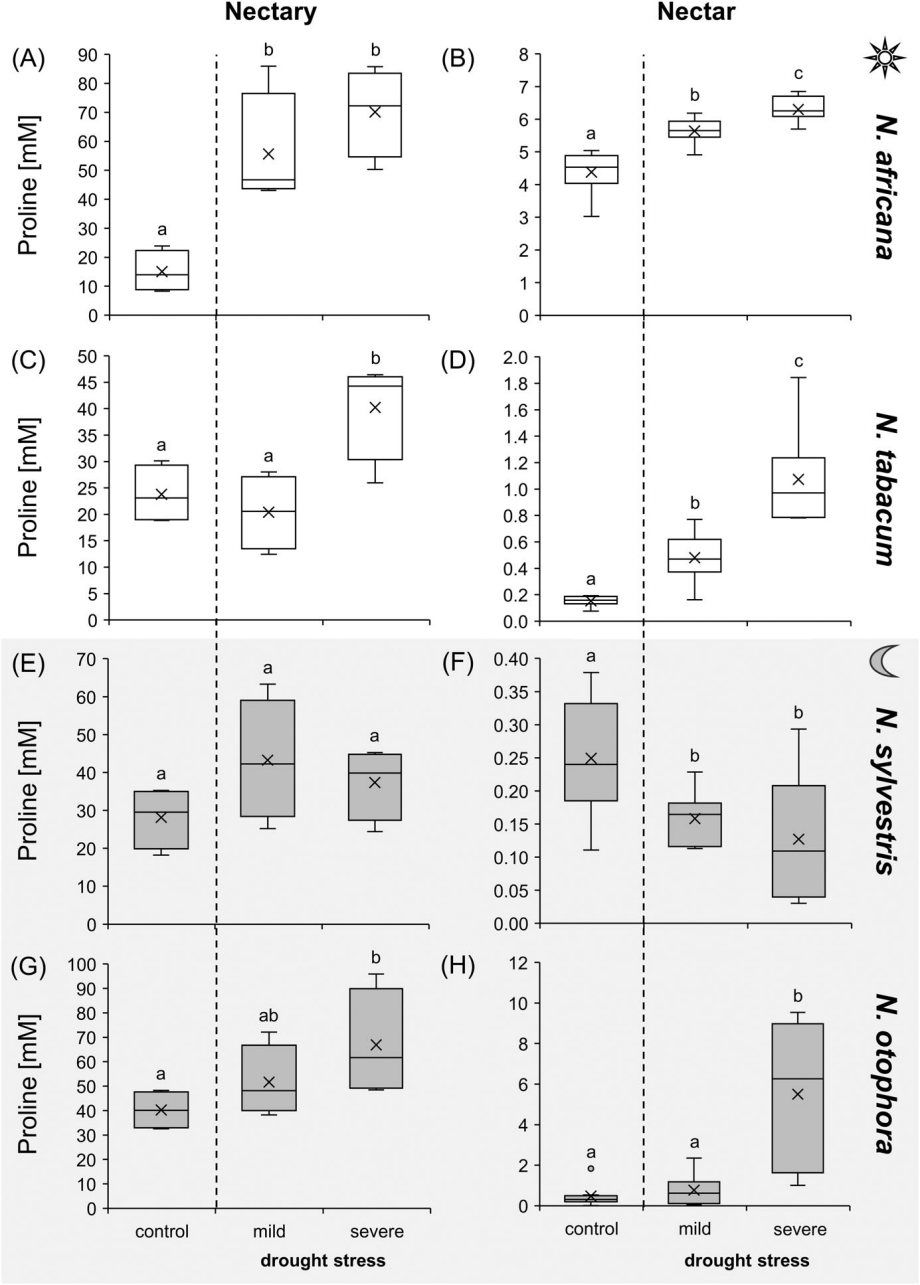
Proline concentration in nectaries and nectar of four *Nicotiana* species under different drought treatments (control, mild, severe). To directly compare proline concentrations in nectaries (A, C, E, G) and nectar (B, D, F, H), both are presented with the same unit. The *Nicotiana* species include 2 day‐flowering (A–D) and two night‐flowering (E–H) species. The day‐flowering species are *N. africana* (A & B) and *N. tabacum* (C & D). The night‐flowering species are *N. sylvestris* (E & F) and *N. otophora* (G & H). Different letters represent significant differences in nectar volume between treatments with drought (Tukey HSD; *P* < 0.05; nectaries *n* = 4; nectar: *n* = 8).

Since proline is produced in increased amounts in some plants under drought stress, this amino acid was also investigated (Fig. 6, Tables S3–S6). Under control conditions, proline in nectaries of *Nicotiana* species was 15 to 40 mM, while in nectar it was ca. 100‐fold lower, below 0.5 mM (Fig. 6). In nectaries of *Nicotiana* species, proline concentration increased, at least under severe drought stress and to varying degrees (Fig. 6A,C,E,G). An increase in proline concentration was also observed in leaves of *Nicotiana* species under drought stress, at least in *N. africana* and *N. tabacum* (Fig. S4). In nectar, the influence of drought stress on proline concentration was not uniform. In nectar of *N. africana*, *N. tabacum* and *N. otophora*, proline concentration increased 1.5‐, 5‐ and 10‐fold, respectively, whereas in *N. sylvestris* under drought stress only a slight decrease was observed (Fig. 6B,D,F,H).

### Inorganic ion concentrations in nectaries and nectar under drought stress

In leaves and nectaries of all *Nicotiana* species, the concentration of inorganic cations (sum of potassium, sodium, magnesium, calcium, ammonium) were higher than for inorganic anions (sum of chloride, phosphate, sulphate and nitrate) (Tables S3–S6). Furthermore, in leaves, nectaries and nectar, potassium was the most abundant cation, and chloride was the most abundant anion (Table S8).

**Fig. 7:**
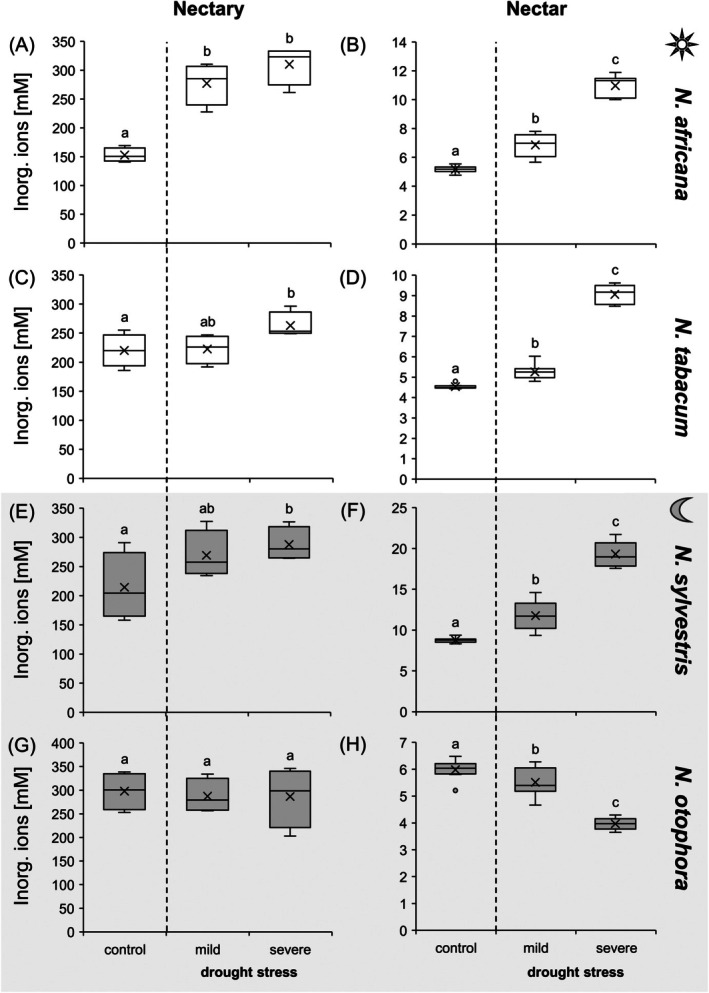
Sum of inorganic ions (cations: potassium, sodium, magnesium, calcium, ammonium; anions: chloride, nitrate, phosphate, sulphate) in nectaries and nectar of four *Nicotiana* species under different drought treatments (control, mild, severe). To directly compare inorganic ion concentrations in nectaries (A, C, E, G) and nectar (B, D, F, H), both are presented with the same unit. The *Nicotiana* species include 2 day‐flowering (A–D) and two night‐flowering (E–H) species. The day‐flowering species are *N. africana* (A & B) and *N. tabacum* (C & D). The night‐flowering species are *N. sylvestris* (E & F) and *N. otophora* (G & H). Different letters represent significant differences in nectar volume between treatments with drought (Tukey's HSD; *P* < 0.05; nectaries *n* = 4; nectar: *n* = 8).

The concentrations of inorganic ions were much higher in the nectaries than in the nectar (Fig. 7), as is the case for amino acids. A significant increase of concentrations of inorganic ions was observed in nectaries of *N. africana*, *N. tabacum* and *N. sylvestris* under severe drought stress (Fig. 7A,C,E). In contrast, no significant changes were found in nectaries of *N. otophora* under drought stress (Fig. 7G). However, it should be noted that concentrations of inorganic ions in nectaries of *N. otophora* were already higher under control conditions than in the other *Nicotiana* species (Fig. 7A,C,E,G). In nectar of *N. africana*, *N. tabacum* and *N. sylvestris* concentrations of inorganic ions also increased under drought stress (Fig. 7B,D,F), whereas in *N. otophora* the concentration decreased during the same period (Fig. 7H). Similar results were found for leaves, where there was a significant increase in concentrations of inorganic ions in all *Nicotiana* species under severe drought stress (Fig. S5). The increase in total inorganic ion concentration in leaves, nectaries and nectar of the four *Nicotiana* species under drought stress was related to an increase in concentrations of various inorganic anions and cations (Table S8).

### Starch content in leaves and nectaries under drought stress

Nectar contained no starch, while nectaries contained 3–9 mg g^−1^ FW (measured as glucose equivalents; Fig. S6, Table S9). Under mild and severe drought stress, starch content showed no significant changes, only in nectaries of *N. tabacum* did starch content increase under severe drought stress. The starch content in leaves was lower than in nectaries and ranged between 1.5 and 3.5 mg g^−1^ FW (Fig. S6). Under drought stress, starch content of leaves showed did not change significantly, only in leaves of *N. africana* did starch content decrease.

### Amount of different nectar compounds per flower under drought stress

Table 1 shows the total amount of sugars, amino acids and inorganic ions in nectar per flower. The amounts were calculated based on nectar concentrations (Figs. 3, 5, 7) and nectar volumes (Fig. 2). In all *Nicotiana* species under drought stress, the total amount of sugar per flower decreased sharply (Table 1). This also applies to total amount of amino acids per flower and total amount of inorganic ions per flower in the different *Nicotiana* species, with the exception of *N. tabacum* (Table 1).

**Table 1:** Amount of different nectar compounds per flower in four *Nicotiana* species under drought stress.

	∑ sugars [μmol flower^−1^]	∑ amino acids [μmol flower^−1^]	∑ inorganic ions [μmol flower^−1^]
*N. africana*			
control	129.1 ± 16.4^a^	1.3 ± 0.3^a^	0.5 ± 0.0^a^
mild	70.6 ± 18.0^b^	1.0 ± 0.3^b^	0.5 ± 0.1^b^
severe	32.0 ± 7.8^c^	0.4 ± 0.1^c^	0.2 ± 0.0^c^
*N. tabacum*			
control	31.2 ± 5.1^a^	0.02 ± 0.00^a^	0.13 ± 0.00^a^
mild	23.0 ± 5.1^b^	0.04 ± 0.01^b^	0.11 ± 0.01^b^
severe	23.8 ± 2.2^b^	0.03 ± 0.01^b^	0.11 ± 0.01^b^
*N. sylvestris*			
control	29.7 ± 3.8^a^	0.03 ± 0.01^a^	0.3 ± 0.0^a^
mild	19.5 ± 4.3^b^	0.01 ± 0.00^b^	0.2 ± 0.0^b^
severe	8.1 ± 1.9^c^	0.00 ± 0.00^b^	0.1 ± 0.0^c^
*N. otophora*			
control	197.4 ± 27.0^a^	0.3 ± 0.2^a^	1.3 ± 0.1^a^
mild	45.6 ± 4.8^b^	0.1 ± 0.1^b^	0.5 ± 0.0^b^
severe	4.7 ± 1.0^c^	0.0 ± 0.0^b^	0.0 ± 0.0^c^

Total amounts per flower were calculated using the nectar concentrations (Figs. 3, 5, 7) and nectar volumes (Fig. 2). All amounts are mean ± SD (*n* = 8) and given in μmol per flower. Different letters represent significant differences in each sum of sugars, amino acids and inorganic ions, between treatments with drought (Tukey HSD; *P* < 0.05).

### Influence of drought treatments on metabolite and ion composition in nectaries and nectar

**Fig. 8:**
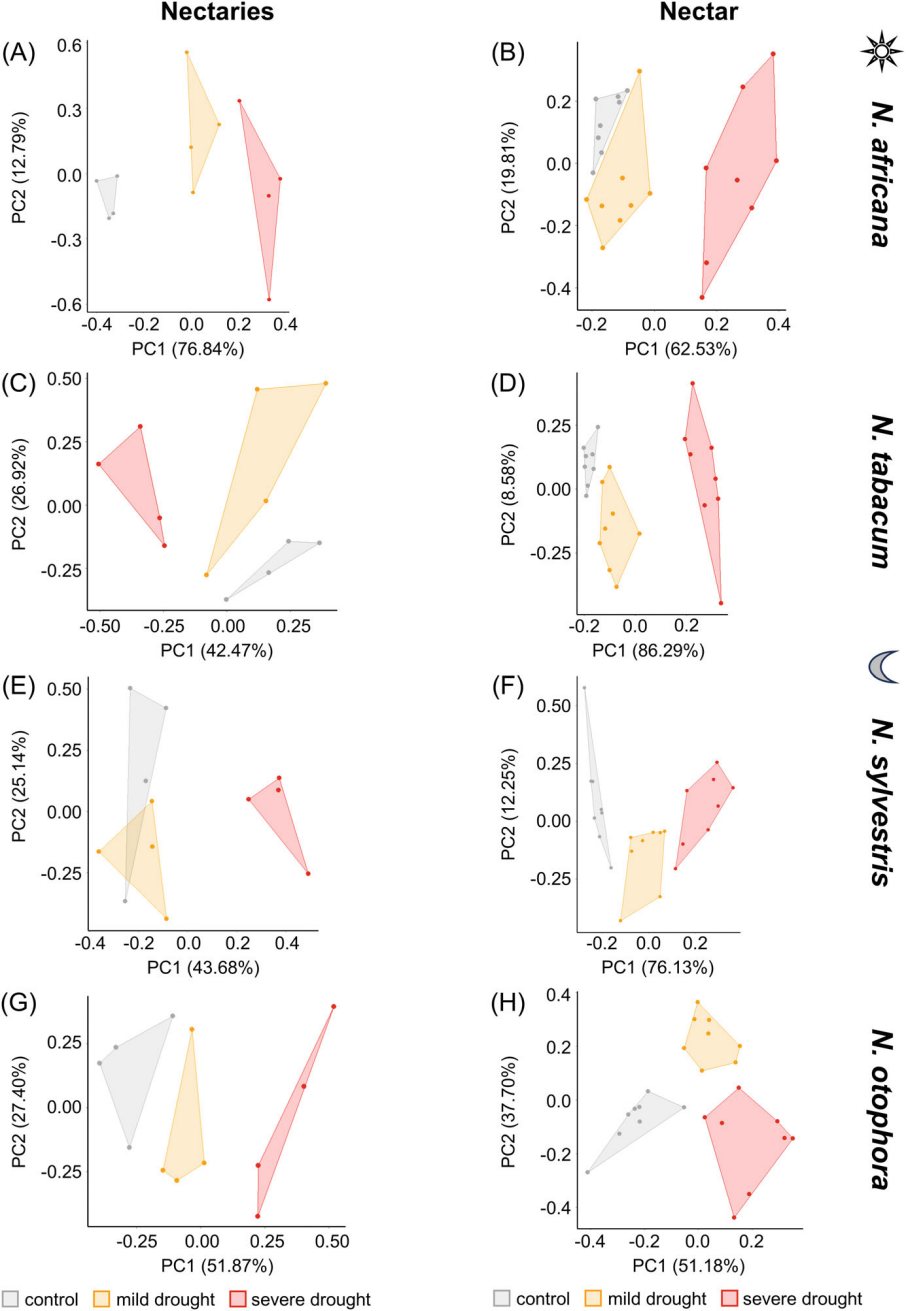
Scatterplots of PCA results for nectary tissue (A, C, E, G) and nectar (B, D, F, H) data from four *Nicotiana* species under different drought treatments. Sum of sugars, amino acids and inorganic ions data in nectaries (*n* = 4) and nectar (*n* = 8) were used for PCA. PCAs are divided into day‐ (A–D) and night‐flowering (E–H) species. PCAs for nectary tissue and nectar data from *N. africana* (A, B), *N. tabacum* (C, D), *N. sylvestris* (E, F), and *N. otophora* (G, H).

A PCA was performed to investigate whether the variance in sugar, amino acid and inorganic ion concentrations in nectaries and nectar of the *Nicotiana* species compared to control plants could be explained by mild and severe drought stress (Fig. 8). In addition, PCA was also performed for leaves (Fig. S7). The PCAs were separated according to *Nicotiana* species and the respective tissue.

The PCA separated plants under drought stress from control plants based on metabolite and ion concentration in leaves (Fig. S7). In addition, with the exception of *N. tabacum*, it was possible to separate plants from the two drought stress phases (Fig. S7A,C,D). The PCA data from leaves of the different species explained 74.77% (*N. tabacum*; Fig. S7B) to 90.08% (*N. africana*; Fig. S7A) of the total variance of the data based and subdivided by drought stress conditions. The PERMANOVA supported graphical evaluation of the PCA. For the four *Nicotiana* species, between 42% and 84% of the data variance in leaves could be explained by the drought stress condition (Table S10; *P* ≤ 0.001).

In both PCAs with nectary data and in those for nectar data of the *Nicotiana* species, control plants and plants with mild or severe drought stress were graphically separated (Fig. 8). When considering data on nectaries of the four *Nicotiana* species in the PCA, between 68.82% (*N. sylvestris*; Fig. 8E) and 89.63% (*N. africana*; Fig. 8A) of the total variance was based on the principal components. This was even more pronounced for PCAs on the nectar data, where 82.34% (*N. africana*; Fig. 8A) and 94.87% (*N. tabacum*; Fig. 8B) of total variance based on principal components could be explained. The PERMANOVA supports graphical evaluation of the PCA. For the four *Nicotiana* species, between 84% and 27% of data variance of nectaries, and between 85% and 40% in nectar can be explained by the level of drought stress (Table 2; *P* ≤ 0.001).

**Table 2:** Results of PERMANOVA and PERMDISP of the day‐ (*N. africana*, *N. tabacum*) and night‐flowering (*N. sylvestris*, *N. otophora*) *Nicotiana* species separated into nectary tissue (A) and nectar (B) data.

	degrees of freedom (df)	pseudo‐F (*F*)	*R* ^2^	PERMANOVA *P*‐value	PERMDISP *P*‐value
*Nicotiana africana*					
(A) Nectary					
drought treatment	2	22.58	0.84	0.001***	0.501
residuals	9		0.16		
total	11		1.00		
(B) Nectar					
drought treatment	2	6.90	0.40	0.008**	0.131
residuals	21		0.60		
total	23		1.00		
*Nicotiana tabacum*					
(A) Nectary					
drought treatment	2	2.66	0.37	0.093	0.594
residuals	9		0.63		
total	11		1.00		
(B) Nectar					
drought treatment	2	48.20	0.85	0.001***	0.224
residuals	21		0.15		
total	23		1.00		
*Nicotiana sylvestris*					
(B) Nectary					
drought treatment	2	5.60	0.55	0.004**	0.725
residuals	9		0.45		
total	11		1.00		
(C) Nectar					
drought treatment	2	10.87	0.51	0.001***	0.041
residuals	21		0.49		
total	23		1.00		
*Nicotiana otophora*					
(B) Nectary					
drought treatment	2	1.65	0.27	0.206	0.754
residuals	9		0.73		
total	11		1.00		
(C) Nectar					
drought treatment	2	21.14	0.67	0.001***	0.101
residuals	21		0.33		
total	23		1.00		

Significance level for PERMDISP is *P* ≤ 0.001; significance level for PERMANOVA: *P < 0.05, **P < 0.01, ***P < 0.001 .

## DISCUSSION

**Fig. 9:**
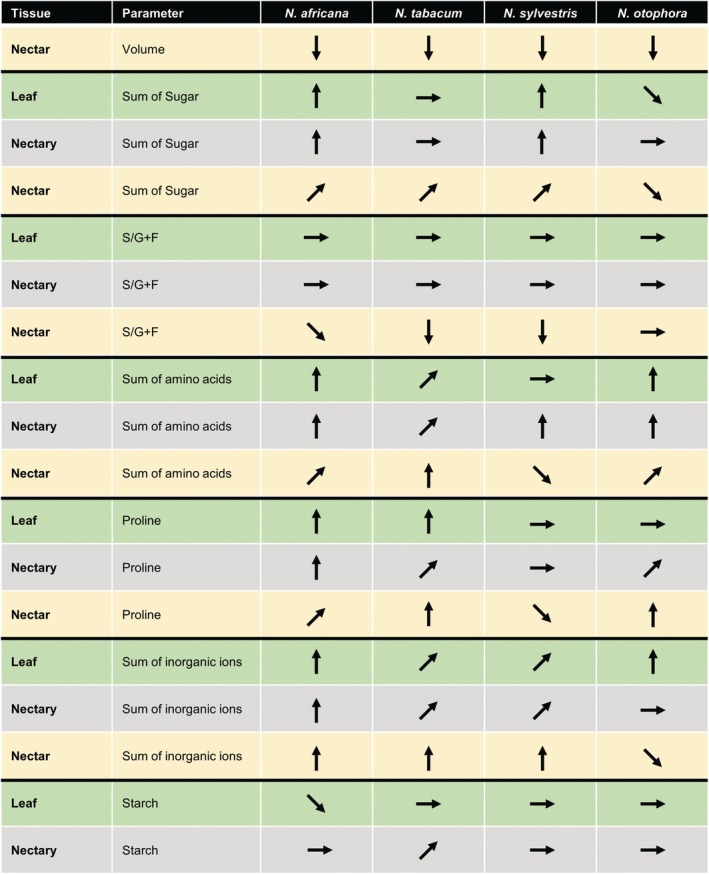
Summary of changes in concentrations of metabolites and inorganic ions in leaves, nectaries and nectar of the four *Nicotiana* species under drought stress, based on results in Figs. 2, 3, 4, 5, 6, 7 (nectaries and nectar) and Figs. S1–S6 (leaves). The different arrows describe: ⮕ no changes to the parameter; ⬈ slight increase; **↑** strong increase; ⬊ slight decrease; **↓** strong decrease.

The nectar composition is relatively consistent for a given species but varies between species, and these differences can be partly attributed to the preferences of the pollinators of a particular species (Baker & Baker 1983; Abrahamczyk *et al*. 2017; Tiedge & Lohaus 2017; Göttlinger *et al*. 2019). The composition of the nectar can also be influenced by environmental conditions, such as drought stress (Clearwater *et al*. 2018; Descamps *et al*. 2021a). This also applies to the *Nicotiana* species studied, since drought stress not only led to altered metabolite composition in leaves, but also to an altered composition of nectar and nectaries (summarized in Fig. 9).

### Drought stress leads to a decrease in nectar volume

In many plant species, drought stress leads to a reduced number of flowers and lower nectar volumes (Kuppler & Kotowska 2021). However, there are individual examples where no change in nectar volume occurred after drought stress, for example in *Lathyrus pratensis* (Phillips *et al*. 2018). Because of the different effects of drought stress on nectar volume, it is assumed that this depends on the plant species (Descamps *et al*. 2021a), the local adaptation of the population (Suni *et al*. 2020) or the type of study, indoor (greenhouse) vs. outdoor (field experiments) experiments (Kuppler & Kotowska 2021). In all four *Nicotiana* species, drought stress resulted in a significant decrease in the number of open flowers per day (Table S2) and in nectar volume, regardless of flowering time or pollination type (Fig. 2). One explanation for the decrease in nectar volume could be evaporation. However, in all *Nicotiana* species examined, the nectar was buried deep within the flowers (Fig. 1). It is more likely that during drought stress the reduced water availability in the plant leads to less water being supplied to the nectar during secretion (Carroll *et al*. 2001; Gallagher & Campbell 2017). It was shown that nectar volume in flowers of different plant species correlates with the size of their pollinators (Tiedge & Lohaus 2017). Therefore, the strong reduction in nectar volume during drought stress in *N. Africana*, and especially in *N. otophora* (about 20‐fold lower; Fig. 2) can severely affect their pollinators, that is sunbirds and bats.

### Influence of drought treatment on metabolite and ion composition in nectaries and nectar

The influence of drought stress on nectar composition, especially related to sugars, have been described in several plant species (Descamps *et al*. 2018; Rering *et al*. 2020; Plos *et al*. 2023), but so far studies on the influence of drought stress on metabolism in nectaries, as well as parallel analyses of nectar and nectaries are rare (Göttlinger & Lohaus 2020). For all four *Nicotiana* species, the compounds in leaves as well as in nectar and nectaries can be used to distinguish control samples from samples under mild or severe drought stress using PCA (Fig. 8, Fig. S7). The graphical analysis was then further supported by PERMANOVA, which confirmed that the data variation present in the nectary samples as well as in nectar samples for all species was significantly related to drought stress (Table 2). Nevertheless, the influence of drought stress on nectar and nectary composition varies among the different *Nicotiana* species, which have different flowering times and pollinators (Figs. 1, 2, 3, 4, 5, 6, 7, Tables S3–S6). In addition, the influence of drought stress on sugars, amino acids or inorganic ions in nectar and nectaries also varied (Fig. 9).

### Comparison of leaf, nectary and nectar composition under drought stress

#### Sugars

Regarding total sugar concentration in the nectaries, there was no increase in two of the *Nicotiana* species (Fig. 3C,G) under severe drought stress, whereas there was a significant increase in the other two species (Fig. 3A,E). The same influence of drought stress was observed on sugar concentrations in leaves of the four *Nicotiana* species (Fig. 9). In the nectar, total sugar concentration increased significantly in three *Nicotiana* species (Fig. 3B,D,F) under drought stress, whereas it decreased in *N. otophora* (night‐flowering, bat‐pollinated; Fig. 3H). There is also no consensus in the literature on the influence of drought stress on sugar concentration in nectar (Borghi *et al*. 2019). While some studies did not find significant changes in nectar sugar concentration during drought stress (Phillips *et al*. 2018; Göttlinger & Lohaus 2020), another reported an increase (Suni *et al*. 2020).

In leaves and nectaries of all *Nicotiana* species, the ratio of sucrose:hexoses remained constant during drought stress, whereas in the nectar, with the exception *N. otophora*, the ratio decreased during the drought period (Fig. 9). A decrease in this ratio in nectar during drought stress was also observed in the bromeliads *Aechmea fasciata* and *Billbergia nutans* (Göttlinger & Lohaus 2020), as well as in *Borago officinalis* (Descamps *et al*. 2021a) and *Fagopyrum esculentum* (Clearwater *et al*. 2018). One possible explanation for the increased hexose concentration in relation to the sucrose concentration in nectar could be that hexose‐rich nectars have a higher osmolality than sucrose‐rich nectars at the same sugar concentration. Higher osmolality of nectar can, in turn, reduces evaporation, which is beneficial in dry conditions (Corbet 1978; Nicolson 1994). The changes in sugar composition of the nectar are not reflected in the nectaries of the *Nicotiana* species. Therefore, the decreased in sucrose:hexoses ratio under drought stress could be the result of increased sucrose cleavage during secretion or altered transport processes, such as increased export of hexoses and decreased export of sucrose from nectaries (Tiedge & Lohaus 2018; Göttlinger & Lohaus 2020). The activity of SWEET9 is generally important for the transport of sucrose from the nectaries into the nectar (Lin *et al*. 2014), but the influence of drought stress on this transport process in nectaries has not yet been analysed.

Although the total sugar concentration in nectar increased in most *Nicotiana* species under drought stress, the total amount of sugars per flower decreased sharply in all *Nicotiana* species due to the strong decrease in nectar volume (Table 1). Furthermore, the number of open flowers per day also decreased in the four *Nicotiana* species under drought stress (Table S2), which again leads to a reduction in nectar supply for pollinators. This was particularly the case in *N. otophora* (pollinated by bats), where the amount of sugar per flower under drought stress was about 40 times lower than in flowers of control plants (Table 1). Nectarivorous bats are particularly at high risk of being affected by climate changes because of their specialized diet (Zamora‐Gutierrez *et al*. 2021). The low amount of sugar per flower, together with the altered sugar composition of the nectar can generally affect the food supply for pollinators and influence their foraging behaviour (Descamps *et al*. 2021b). In addition, higher sugar concentrations in nectar can also have negative impact on nectar uptake by pollinators, as the viscosity of the nectar increases with sugar concentration, making it more difficult for pollinators to extract (Kim *et al*. 2011).

#### Amino acids

Compared to nectar, the total amino acid concentration in nectaries was generally much higher (5‐ to 75‐fold; Fig. 5), suggesting that amino acids are retained in nectaries and their export is strongly regulated during nectar secretion (Göttlinger & Lohaus 2022). Significantly higher amino acid concentrations were also found in nectaries of various bromeliad species than in their nectar (Göttlinger & Lohaus 2022, 2023). In general, nectaries are supplied with amino acids from the phloem, but some amino acids can also be produced in the nectaries themselves (Solhaug *et al*. 2021). However, it is not yet fully understood how amino acids are excreted from the nectaries into the nectar, and corresponding transporters have not yet been identified (Borghi & Fernie 2017; Nicolson 2022).

Under drought stress, total amino acid concentration increased in leaves, nectaries and nectar of all *Nicotiana* species, except for the nectar of *N. sylvestris* (Fig. 9). An increase in the amino acid concentration was also observed in the nectar of *Borago officinalis* (Descamps *et al*. 2021a). Although the total amino acid concentration in nectar increased under drought stress (Fig. 4), the total amount of amino acids per flower decreased, with the exception of *N. tabacum*, because the nectar volume decreased sharply at the same time (Fig. 2, Table 1). The low amount of amino acids at the flower level can impair food supply for pollinators and influence their foraging behaviour (Descamps *et al*. 2021b). This influence may vary depending on whether pollinators rely exclusively on nectar for their nitrogen uptake or whether they can use pollen or other alternative protein sources (Baker & Baker 1973). In addition, the nitrogen requirement and the body size of the pollinator also play a role (Tiedge & Lohaus 2017).

The amino acid proline is a compatible osmolyte with several functions in plants, such as stabilizing membranes and scavenging reactive oxygen species (Liang *et al*. 2013). Several studies have shown that proline content increases in various plant tissues in response to stress, including drought stress (Hayat *et al*. 2012). Proline concentrations also increased in the leaves, nectaries and nectar of the *Nicotiana* species under drought stress, at least in *N. africana*, *N. tabacum* and *N. otophora* (Fig. 9).

The amino acid composition in nectar is also related to preferences of the pollinators (Baker & Baker 1973; Tiedge & Lohaus 2017). Honeybees, for example, prefer artificial nectars that are rich in proline (Carter *et al*. 2006) and hummingbirds may also be attracted by proline in nectar (Quintana‐Rodríguez *et al*. 2018). The amino acid content can also influence the food selection of hawkmoths and bats (Rodríguez‐Peña *et al*. 2013; Broadhead & Raguso 2021), but a preference for proline or other amino acids is not yet resolved.

#### Inorganic ions

Similar to amino acids, the concentration of inorganic ions in nectaries was much higher than in the nectar of *Nicotiana* species (Fig. 7). This difference between nectaries and nectar has already been observed in several bromeliad species (Göttlinger & Lohaus 2020, 2022). With the exception of *N. otophora*, the total ion concentration increased in both the nectaries and nectar during drought stress in the *Nicotiana* species (Fig. 7). Here, too, the total concentration of inorganic ions in the nectar increased under drought stress (Fig. 7), but the total amount of inorganic ions per flower decreased because there was also a large reduction in nectar volume (Fig. 2, Table 1). However, how these changes affect pollinators must be investigated in future studies.

## CONCLUSION

Because the frequency of droughts will increase worldwide in the future, it is necessary to investigate the influence on plant growth and reproduction, as well as on plant–pollinator interactions. Until now, the composition of nectaries and nectar under drought stress has received little attention. This is the first time that different plant species with different pollinators, including sunbirds and bats, have been included in such studies. The four *Nicotiana* species analysed responded to drought stress with reduced nectar volume and changes in the metabolite composition of both nectar and nectaries. While the day‐flowering species (pollinated by sunbirds or hummingbirds) showed relatively similar changes, the night‐flowering species (pollinated by hawkmoths or bats) responded differently to plant drought stress. In all *Nicotiana* species, the total amount of sugars, amino acids and inorganic ions per flower decreased sharply because of the strong decrease in nectar volume. These changes can disrupt interactions between plant and pollinator. However, further research is needed to understand how different plant species respond to drought stress, especially considering the different pollinators of plant species.

## AUTHOR CONTRIBUTIONS

GL designed the research and acquired the funding. TG helped with the methodology. DN performed the research and investigation. TG, DN and JD analysed the data. TG and DN performed the graphic visualization. GL, TG and DN wrote the manuscript. TG and DN contributed equally to this work. All authors contributed, read and approved the final manuscript.

## FUNDING INFORMATION

This work was supported by the Deutsche Forschungsgemeinschaft (LO 734/10‐2).

## Supporting information


**Table S1.** Water content of leaves, expressed as percentage of fresh weight.
**Table S2.** Number of open flowers per day and plant of control plants and plants under severe drought stress.
**Table S3.** Metabolic data of *Nicotiana africana* under control and different drought stress conditions.
**Table S4.** Metabolic data of *Nicotiana tabacum* under control and different drought stress conditions.
**Table S5.** Metabolic data of *Nicotiana sylvestris* under control and different drought stress conditions.
**Table S6.** Metabolic data of *Nicotiana otophora* under control and different drought stress conditions.
**Table S7.** Concentrations of various amino acids in leaves, nectaries and nectar of the four *Nicotiana* species under control and different drought stress conditions.
**Table S8.** Concentrations of various inorganic ions in leaves, nectaries and nectar under different drought treatments in *Nicotiana* species.
**Table S9.** Starch content of leaves and nectaries of different *Nicotiana* species.
**Table S10.** Results of PERMANOVA and PERMDISP of day‐ and night‐flowering *Nicotiana* species separated to leaf data.
**Fig. S1.** Sugar concentrations in leaves of four *Nicotiana* species under different drought treatments (control, mild, severe).
**Fig. S2.** Sucrose‐to‐hexoses ratio (ref. mM) in leaves of four *Nicotiana* species under different drought treatments (control, mild, severe).
**Fig. S3.** Amino acid concentrations in leaves of four *Nicotiana* species under different drought treatments (control, mild, severe).
**Fig. S4.** Proline concentrations in leaves of four *Nicotiana* species under different drought treatments (control, mild, severe).
**Fig. S5.** Inorganic ion concentrations in leaves of four *Nicotiana* species under different drought treatments (control, mild, severe).
**Fig. S6.** Starch content measured as mg glucose equivalents g^−1^ FW in leaves (A, C, E, G) and nectaries (B, D, F, H) of four *Nicotiana* species under different drought treatments (control, mild, severe).
**Fig. S7.** Scatterplots of PCA for leaf data from four *Nicotiana* species of different drought treatments.

## References

[plb70000-bib-0001] Abrahamczyk S. , Kessler M. , Hanley D. , Karger D.N. , Müller M.P.J. , Knauer A.C. , Keller F. , Schwerdtfeger M. , Humphreys A.M. (2017) Pollinator adaptation and the evolution of floral nectar sugar composition. Journal of Evolutionary Biology, 30, 112–127. 27747987 10.1111/jeb.12991

[plb70000-bib-0002] Anderson M.J. (2006) Distance‐based tests for homogeneity of multivariate dispersions. Biometrics, 62, 245–253. 16542252 10.1111/j.1541-0420.2005.00440.x

[plb70000-bib-0003] Anderson M.J. (2014) Permutational multivariate analysis of variance (PERMANOVA). In: Balakrishnan N. , Colton T. , Everitt B. , Piegorsch W. , Ruggeri F. , Teugels J.L. (Eds), Wiley StatsRef: statistics reference online. Wiley, New York, USA, pp 1–15.

[plb70000-bib-0004] Baker H.G. , Baker I. (1973) Amino‐acids in nectar and their evolutionary significance. Nature, 241, 543–545.4693956

[plb70000-bib-0005] Baker H.G. , Baker I. (1983) Floral nectar sugar constituents in relation to pollinator type. In: Jones C.E. , Little R.J. (Eds), Handbook of experimental pollination biology. Van Nostrand Reinhold, New York, pp 117–141.

[plb70000-bib-0006] Borghi M. , de Souza L.P. , Yoshida T. , Fernie A.R. (2019) Flowers and climate change. A metabolic perspective. New Phytologist, 224, 1425–1441. 31257600 10.1111/nph.16031

[plb70000-bib-0007] Borghi M. , Fernie A.R. (2017) Floral metabolism of sugars and amino acids: Implications for pollinators' preferences and seed and fruit set. Plant Physiology, 175, 1510–1524. 28986424 10.1104/pp.17.01164 PMC5717749

[plb70000-bib-0008] Broadhead G.T. , Raguso R.A. (2021) Associative learning of non‐sugar nectar components: amino acids modify nectar preference in a hawkmoth. Journal of Experimental Biology, 224, jeb234633. 34142140 10.1242/jeb.234633 PMC8246342

[plb70000-bib-0009] Brown M.J.F. , Dicks L.V. , Paxton R.J. , Baldock K.C.R. , Barron A.B. , Chauzat M.‐P. , Freitas B.M. , Goulson D. , Jepsen S. , Kremen C. , Li J. , Neumann P. , Pattemore D.E. , Potts S.G. , Schweiger O. , Seymour C.L. , Stout J.C. (2016) A horizon scan of future threats and opportunities for pollinators and pollination. PeerJ, 4, e2249. 27602260 10.7717/peerj.2249 PMC4991895

[plb70000-bib-0010] Carroll A.B. , Pallardy S.G. , Galen C. (2001) Drought stress, plant water status, and floral trait expression in fireweed, *Epilobium angustifolium* (Onagraceae). American Journal of Botany, 88, 438–446. 11250821

[plb70000-bib-0011] Carter C. , Graham R.A. , Thornburg R.W. (1999) Nectarin I is a novel, soluble germin‐like protein expressed in the nectar of *Nicotiana* sp. Plant Molecular Biology, 41, 207–216. 10579488 10.1023/a:1006363508648

[plb70000-bib-0012] Carter C. , Shafir S. , Yehonatan L. , Palmer R.G. , Thornburg R. (2006) A novel role for proline in plant floral nectars. Naturwissenschaften, 93, 72–79. 16365739 10.1007/s00114-005-0062-1

[plb70000-bib-0013] Chase M.W. , Paun O. , Fay M.F. (2010) Hybridization and speciation in angiosperms: a role for pollinator shifts? BMC Biology, 8, 45. 20409350 10.1186/1741-7007-8-45 PMC2858105

[plb70000-bib-0014] Ciais P. , Reichstein M. , Viovy N. , Granier A. , Ogée J. , Allard V. , Aubinet M. , Buchmann N. , Bernhofer C. , Carrara A. , Chevallier F. , deNoblet N. , Friend A.D. , Friedlingstein P. , Grünwald T. , Heinesch B. , Keronen P. , Knohl A. , Krinner G. , Loustau D. , Manca G. , Matteucci G. , Miglietta F. , Ourcival J.M. , Papale D. , Pilegaard K. , Rambal S. , Seufert G. , Soussana J.F. , Sanz M.J. , Schulze E.D. , Vesala T. , Valentini R. (2005) Europe‐wide reduction in primary productivity caused by the heat and drought in 2003. Nature, 437, 529–533. 16177786 10.1038/nature03972

[plb70000-bib-0015] Clearwater M.J. , Revell M. , Noe S. , Manley‐Harris M. (2018) Influence of genotype, floral stage, and water stress on floral nectar yield and composition of mānuka (*Leptospermum scoparium*). Annals of Botany, 121, 501–512. 29300875 10.1093/aob/mcx183 PMC5838834

[plb70000-bib-0016] Corbet S.A. (1978) Bee visits and the nectar of *Echium vulgare* L. and *Sinapis alba* L. Ecological Entomology, 3, 25–37.

[plb70000-bib-0017] Dai A. (2013) Increasing drought under global warming in observations and models. Nature Climate Change, 3, 52–58.

[plb70000-bib-0018] Descamps C. , Quinet M. , Baijot A. , Jacquemart A.‐L. (2018) Temperature and water stress affect plant–pollinator interactions in *Borago officinalis* (Boraginaceae). Ecology and Evolution, 8, 3443–3456. 29607037 10.1002/ece3.3914 PMC5869376

[plb70000-bib-0019] Descamps C. , Quinet M. , Jacquemart A.‐L. (2021a) Climate change‐induced stress reduces quantity and alters composition of nectar and pollen from a bee‐pollinated species (*Borago officinalis*, Boraginaceae). Frontiers in Plant Science, 12, 755843. 34707633 10.3389/fpls.2021.755843 PMC8542702

[plb70000-bib-0020] Descamps C. , Quinet M. , Jacquemart A.‐L. (2021b) The effects of drought on plant–pollinator interactions: What to expect? Environmental and Experimental Botany, 182, 104297.

[plb70000-bib-0021] Dietz K.‐J. , Zörb C. , Geilfus C.‐M. (2021) Drought and crop yield. Plant Biology, 23, 881–893. 34396653 10.1111/plb.13304

[plb70000-bib-0022] Fahn A. (1979) Ultrastructure of nectaries in relation to nectar secretion. American Journal of Botany, 66, 977–985.

[plb70000-bib-0023] Gallagher M.K. , Campbell D.R. (2017) Shifts in water availability mediate plant–pollinator interactions. New Phytologist, 215, 792–802. 28517023 10.1111/nph.14602

[plb70000-bib-0024] Gautam S. , Mishra U. , Scown C.D. , Ghimire R. (2023) Increased drought and extreme events over continental United States under high emissions scenario. Scientific Reports, 13, 21503. 38057376 10.1038/s41598-023-48650-z PMC10700340

[plb70000-bib-0025] Göttlinger T. , Lohaus G. (2020) Influence of light, dark, temperature and drought on metabolite and ion composition in nectar and nectaries of an epiphytic bromeliad species (*Aechmea fasciata*). Plant Biology, 22, 781–793. 32558085 10.1111/plb.13150

[plb70000-bib-0026] Göttlinger T. , Lohaus G. (2022) Comparative analyses of the metabolite and ion concentrations in nectar, nectaries, and leaves of 36 bromeliads with different photosynthesis and pollinator types. Frontiers in Plant Science, 13, 987145. 36092434 10.3389/fpls.2022.987145 PMC9459329

[plb70000-bib-0027] Göttlinger T. , Lohaus G. (2023) Origin and function of amino acids in nectar and nectaries of *Pitcairnia* species with particular emphasis on alanine and glutamine. Plants, 13, 23. 38202331 10.3390/plants13010023 PMC10780904

[plb70000-bib-0028] Göttlinger T. , Schwerdtfeger M. , Tiedge K. , Lohaus G. (2019) What do nectarivorous bats like? Nectar composition in Bromeliaceae with special emphasis on bat‐pollinated species. Frontiers in Plant Science, 10, 205. 30847001 10.3389/fpls.2019.00205 PMC6393375

[plb70000-bib-0029] Halpern S.L. , Adler L.S. , Wink M. (2010) Leaf herbivory and drought stress affect floral attractive and defensive traits in *Nicotiana quadrivalvis* . Oecologia, 163, 961–971. 20461411 10.1007/s00442-010-1651-z

[plb70000-bib-0030] Hayat S. , Hayat Q. , Alyemeni M.N. , Wani A.S. , Pichtel J. , Ahmad A. (2012) Role of proline under changing environments: a review. Plant Signaling & Behavior, 7, 1456–1466. 22951402 10.4161/psb.21949 PMC3548871

[plb70000-bib-0031] Kaczorowski R.L. , Gardener M.C. , Holtsford T.P. (2005) Nectar traits in *Nicotiana* section Alatae (Solanaceae) in relation to floral traits, pollinators, and mating system. American Journal of Botany, 8, 1270–1283. 10.3732/ajb.92.8.1270 21646148

[plb70000-bib-0032] Kessler D. , Bhattacharya S. , Diezel C. , Rothe E. , Gase K. , Schöttner M. , Baldwin I.T. (2012) Unpredictability of nectar nicotine promotes outcrossing by hummingbirds in *Nicotiana attenuata* . Plant Journal for Cell and Molecular Biology, 71, 529–538. 22448647 10.1111/j.1365-313X.2012.05008.x

[plb70000-bib-0033] Kim W. , Gilet T. , Bush J.W. (2011) Optimal concentrations in nectar feeding. Proceedings of the National Academy of Sciences, USA, 108, 16618–16621. 10.1073/pnas.1108642108 PMC3189050 21949358

[plb70000-bib-0034] Kuppler J. , Kotowska M.M. (2021) A meta‐analysis of floral trait responses and flower‐visitor interaction to water deficit. Global Change Biology, 27, 3095–3108. 33774883 10.1111/gcb.15621

[plb70000-bib-0035] Kuppler J. , Wieland J. , Junker R.R. , Ayasse M. (2021) Drought‐induced reduction in flower size and abundance correlates with reduced flower visits by bumble bees. AoB Plants, 13, plab001. 33628409 10.1093/aobpla/plab001 PMC7891244

[plb70000-bib-0036] Liang X. , Zhang L. , Natarajan S.K. , Becker D.F. (2013) Proline mechanisms of stress survival. Antioxidants & Redox Signaling, 19, 998–1011. 23581681 10.1089/ars.2012.5074 PMC3763223

[plb70000-bib-0037] Lin I.W. , Sosso D. , Chen L.‐Q. , Gase K. , Kim S.‐G. , Kessler D. , Klinkenberg P.M. , Gorder M.K. , Hou B.‐H. , Qu X.‐Q. , Carter C.J. , Baldwin I.T. , Frommer W.B. (2014) Nectar secretion requires sucrose phosphate synthases and the sugar transporter SWEET9. Nature, 508, 546–549. 24670640 10.1038/nature13082

[plb70000-bib-0038] Lohaus G. , Pennewiss K. , Sattelmacher B. , Hussmann M. , Hermann M.K. (2001) Is the infiltration‐centrifugation technique appropriate for the isolation of apoplastic fluid? A critical evaluation with different plant species. Physiologia Plantarum, 111, 457–465. 11299010 10.1034/j.1399-3054.2001.1110405.x

[plb70000-bib-0039] Lohaus G. , Schwerdtfeger M. (2014) Comparison of sugars, iridoid glycosides and amino acids in nectar and phloem sap of *Maurandya barclayana*, *Lophospermum erubescens*, and *Brassica napus* . PLoS One, 9, e87689. 24489951 10.1371/journal.pone.0087689 PMC3906183

[plb70000-bib-0040] Lunn J. , Hatch M. (1995) Primary partitioning and storage of photosynthate in sucrose and starch in leaves of C4 plants. Planta, 197, 385–391.

[plb70000-bib-0041] Marlin D. , Nicolson S.W. , Yusuf A.A. , Stevenson P.C. , Heyman H.M. , Krüger K. (2014) The only African wild tobacco, *Nicotiana africana*: alkaloid content and the effect of herbivory. PLoS One, 9, e102661. 25025217 10.1371/journal.pone.0102661 PMC4099186

[plb70000-bib-0042] Nadwodnik J. , Lohaus G. (2008) Subcellular concentrations of sugar alcohols and sugars in relation to phloem translocation in *Plantago major*, *Plantago maritima*, *Prunus persica*, and *Apium graveolens* . Planta, 227, 1079–1089. 18188589 10.1007/s00425-007-0682-0 PMC2270920

[plb70000-bib-0043] Nattero J. , Mor M. , Srsic A.N. , Cocucci A.A. (2003) Possible tobacco progenitors share long‐tongued hawkmoths as pollen vectors. Plant Systematics and Evolution, 241, 47–54.

[plb70000-bib-0044] Nicolson S.W. (1994) Eucalyptus nectar: Production, availability, composition and osmotic consequences for the larva of the eucalypt nectar fly, *Drosophila flavohirta* . South African Journal of Science, 90, 75–79.

[plb70000-bib-0045] Nicolson S.W. (2022) Sweet solutions: nectar chemistry and quality. Philosophical Transactions of the Royal Society of London. Series B, Biological Sciences, 377, 20210163. 35491604 10.1098/rstb.2021.0163 PMC9058545

[plb70000-bib-0046] Phillips B.B. , Shaw R.F. , Holland M.J. , Fry E.L. , Bardgett R.D. , Bullock J.M. , Osborne J.L. (2018) Drought reduces floral resources for pollinators. Global Change Biology, 24, 3226–3235. 29652102 10.1111/gcb.14130

[plb70000-bib-0047] Plos C. , Stelbrink N. , Römermann C. , Knight T.M. , Hensen I. (2023) Abiotic conditions affect nectar properties and flower visitation in four herbaceous plant species. Flora, 303, 152279.

[plb70000-bib-0048] Quintana‐Rodríguez E. , Ramírez‐Rodríguez A.G. , Ramírez‐Chávez E. , Molina‐Torres J. , Camacho‐Coronel X. , Esparza‐Claudio J. , Heil M. , Orona‐Tamayo D. (2018) Biochemical traits in the flower lifetime of a Mexican mistletoe parasitizing mesquite biomass. Frontiers in Plant Science, 9, 1031. 30174673 10.3389/fpls.2018.01031 PMC6108335

[plb70000-bib-0049] Rering C.C. , Franco J.G. , Yeater K.M. , Mallinger R.E. (2020) Drought stress alters floral volatiles and reduces floral rewards, pollinator activity, and seed set in a global plant. Ecosphere, 11, e03254.

[plb70000-bib-0050] Rodríguez‐Peña N. , Stoner K.E. , Ayala‐Berdon J. , Flores‐Ortiz C.M. , Duran A. , Schondube J.E. (2013) Nitrogen and amino acids in nectar modify food selection of nectarivorous bats. Journal of Animal Ecology, 82, 1106–1115. 23550633 10.1111/1365-2656.12069

[plb70000-bib-0051] Seo P.J. , Wielsch N. , Kessler D. , Svatos A. , Park C.‐M. , Baldwin I.T. , Kim S.‐G. (2013) Natural variation in floral nectar proteins of two *Nicotiana attenuata* accessions. BMC Plant Biology, 13, 101. 23848992 10.1186/1471-2229-13-101 PMC3728157

[plb70000-bib-0052] Solhaug E.M. , Roy R. , Venterea R.T. , Carter C.J. (2021) The role of alanine synthesis and nitrate‐induced nitric oxide production during hypoxia stress in *Cucurbita pepo* nectaries. The Plant Journal, 105, 580–599. 33119149 10.1111/tpj.15055

[plb70000-bib-0053] Suni S.S. , Ainsworth B. , Hopkins R. (2020) Local adaptation mediates floral responses to water limitation in an annual wildflower. American Journal of Botany, 107, 209–218. 32080832 10.1002/ajb2.1434

[plb70000-bib-0054] Tiedge K. , Lohaus G. (2017) Nectar sugars and amino acids in day‐ and night‐flowering *Nicotiana* species are more strongly shaped by pollinators' preferences than organic acids and inorganic ions. PLoS One, 12, e0176865. 28467507 10.1371/journal.pone.0176865 PMC5415175

[plb70000-bib-0055] Tiedge K. , Lohaus G. (2018) Nectar sugar modulation and cell wall invertases in the nectaries of day‐ and night‐flowering *Nicotiana* . Frontiers in Plant Science, 9, 622. 29868078 10.3389/fpls.2018.00622 PMC5954170

[plb70000-bib-0056] Waser N.M. , Price M.V. (2016) Drought, pollen and nectar availability, and pollination success. Ecology, 97, 1400–1409. 27459771 10.1890/15-1423.1

[plb70000-bib-0057] Zamora‐Gutierrez V. , Rivera‐Villanueva A.N. , Martínez Balvanera S. , Castro‐Castro A. , Aguirre‐Gutiérrez J. (2021) Vulnerability of bat–plant pollination interactions due to environmental change. Global Change Biology, 27, 3367–3382. 33749983 10.1111/gcb.15611

