# Supplementary material for: Influence of drought stress on the metabolite and ion composition in nectar and nectaries of different day‐ and night‐flowering Nicotiana species
Source: Plant Biol (Stuttg). 2025 Feb 18;28(3):897–912. doi: 10.1111/plb.70000 (PMC13089589; doi:10.1111/plb.70000)
Supplement: Supplementary file 1 — Table S1. Water content of leaves, expressed as percentage of fresh weight. Table S2. Number of open flowers per day and plant of control plants and plants under severe drought stress. Table S3. Metabolic data of Nicotiana africana under control and different drought stress conditions. Table S4. Metabolic data of Nicotiana tabacum under control and different drought stress conditions. Table S5. Metabolic data of Nicotiana sylvestris under control and different drought stress conditions. Table S6. Metabolic data of Nicotiana otophora under control and different drought stress conditions. Table S7. Concentrations of various amino acids in leaves, nectaries and nectar of the four Nicotiana species under control and different drought stress conditions. Table S8. Concentrations of various inorganic ions in leaves, nectaries and nectar under different drought treatments in Nicotiana species. Table S9. Starch content of leaves and nectaries of different Nicotiana species. Table S10. Results of PERMANOVA and PERMDISP of day‐ and night‐flowering Nicotiana species separated to leaf data. Fig. S1. Sugar concentrations in leaves of four Nicotiana species under different drought treatments (control, mild, severe). Fig. S2. Sucrose‐to‐hexoses ratio (ref. mM) in leaves of four Nicotiana species under different drought treatments (control, mild, severe). Fig. S3. Amino acid concentrations in leaves of four Nicotiana species under different drought treatments (control, mild, severe). Fig. S4. Proline concentrations in leaves of four Nicotiana species under different drought treatments (control, mild, severe). Fig. S5. Inorganic ion concentrations in leaves of four Nicotiana species under different drought treatments (control, mild, severe). Fig. S6. Starch content measured as mg glucose equivalents g−1 FW in leaves (A, C, E, G) and nectaries (B, D, F, H) of four Nicotiana species under different drought treatments (control, mild, severe). Fig. S7. Scatterplots of PCA for leaf data f [file PLB-28-897-s001.pdf]

Supplementary Material

Influence of drought stress on the metabolite and ion composition in nectar and nectaries of different day- and night-flowering Nicotiana species

Author: Thomas Göttlinger, Dustin Naegel, Jonathan Emil Dick, Gertrud Lohaus*

*Correspondence: Gertrud Lohaus (lohaus@uni-wuppertal.de)

Supplementary Table S1: Water content in leaves, expressed as a percentage of fresh weight. The water content in leaves was measured under different treatments (control and severe drought stress; n=3). Different letters present significant differences between the two treatments (*t*-test; $p < 0.05$).

Species	control [%]	severe drought stress [%]
Nicotiana africana	85.7 ± 0.8[a]	76.7 ± 1.9[b]
Nicotiana tabacum	85.6 ± 1.6[a]	80.1 ± 0.8[b]
Nicotiana sylvestris	85.4 ± 0.4[a]	70.6 ± 2.5[b]
Nicotiana otophora	84.7 ± 1.3[a]	68.7 ± 0.4[b]

Supplementary Table S2: Number of opened flowers per day and plant of control plants and plants under severe drought treatment (control and severe drought stress; n=4). Different letters present significant differences between the two treatments (*t*-test; $p < 0.05$).

	Number of opened flowers per plant	
**Species**	**control**	**severe drought stress**
Nicotiana africana	9.8 ± 2.8^{a}	3.0 ± 0.8^{b}
Nicotiana tabacum	4.8 ± 1.0^{a}	2.0 ± 1.2^{b}
Nicotiana sylvestris	7.8 ± 1.7^{a}	3.3 ± 1.3^{b}
Nicotiana otophora	9.0 ± 2.2^{a}	3.0 ± 2.2^{b}

Supplementary Table S3: Metabolic data of *Nicotiana africana* under control conditions and different drought stress. The data of leaves (A), nectaries (B), and nectar (C) are presented in different tables.

(A) Leaves of *Nicotiana africana*

Treatment	Glucose [mM]	Fructose [mM]	Sucrose [mM]	Sum amino acids [mM]	Proline [mM]	Sum anions [mM]	Sum cations [mM]
control	35.9	27.5	43.3	7.6	0.7	168.6	270.6
control	109.7	98.8	43.7	5.3	0.8	167.7	270.0
control	30.8	39.8	31.2	4.2	1.8	189.8	329.6
control	43.4	42.8	25.7	5.3	1.6	197.0	412.9
mild	41.0	43.8	59.4	23.7	1.8	293.9	469.9
mild	19.4	25.7	24.5	32.8	7.6	250.7	458.6
mild	44.1	31.6	22.7	34.2	5.2	212.8	755.5
mild	41.9	45.5	29.7	12.2	4.1	265.1	473.7
severe	97.9	95.3	49.8	37.5	5.1	355.1	1152.1
severe	66.1	64.4	53.8	41.4	7.0	439.9	1059.3
severe	27.0	23.8	26.4	38.9	4.8	377.9	1046.5
severe	86.2	95.8	40.7	47.6	7.0	250.3	782.8

(B) Nectaries of *Nicotiana africana*

Treatment	Glucose [mM]	Fructose [mM]	Sucrose [mM]	Sum amino acids [mM]	Proline [mM]	Sum anions [mM]	Sum cations [mM]
control	127.4	153.7	117.8	63.5	10.4	8.8	144.6
control	49.4	64.3	88.2	69.4	17.5	10.0	137.4
control	91.9	74.3	127.6	76.5	23.9	13.2	127.5
control	78.7	89.2	104.9	79.5	8.3	12.6	156.5
mild	83.2	89.1	164.5	99.7	43.1	66.7	209.1
mild	119.0	142.9	124.6	135.6	85.9	42.1	185.7
mild	123.0	143.3	107.4	119.2	45.3	71.6	239.0
mild	95.0	132.5	103.8	113.3	48.2	23.6	271.4
severe	145.7	206.6	159.0	130.6	76.8	54.9	278.4
severe	222.4	300.0	268.4	142.3	50.3	57.0	257.4
severe	200.0	265.4	251.7	156.0	67.7	68.1	264.3
severe	279.0	277.1	181.4	162.2	85.7	80.0	181.5

(C) Nectar of *Nicotiana africana*

Treatment	Glucose [mM]	Fructose [mM]	Sucrose [mM]	Sum amino acids [mM]	Proline [mM]	Sum anions [mM]	Sum cations [mM]
control	527.6	538.6	15.8	11.0	4.4	1.7	3.8
control	689.1	717.6	24.7	14.7	3.0	1.8	3.5
control	561.6	451.5	20.6	15.6	5.0	1.9	3.2
control	689.1	717.6	24.7	17.2	4.5	2.0	3.2
control	732.7	724.4	32.0	10.2	4.6	1.5	3.3
control	677.4	625.0	48.3	17.3	3.9	1.8	3.2
control	645.6	647.5	18.7	11.9	4.5	1.7	3.4
control	563.1	699.6	22.2	11.0	5.0	1.7	3.7
mild	306.6	781.6	11.7	14.0	5.4	1.7	6.1
mild	691.3	999.1	12.0	15.6	6.0	1.6	4.3
mild	330.3	697.2	5.6	11.6	4.9	1.9	5.7
mild	565.4	288.7	6.1	12.9	5.7	1.4	4.2
mild	441.3	458.9	9.9	20.4	5.5	1.5	4.9
mild	709.2	189.0	16.3	8.9	5.8	1.6	5.9
mild	449.7	504.4	11.1	11.6	6.2	1.8	4.6
mild	467.7	514.5	9.3	20.1	5.6	2.3	5.2
severe	702.8	906.7	5.1	16.6	6.3	2.9	7.1
severe	1057.9	1028.2	5.2	20.0	6.2	3.2	6.9
severe	510.3	511.3	6.5	14.0	6.1	4.0	7.5
severe	890.6	287.4	6.2	15.5	5.7	3.6	7.6
severe	654.4	868.8	6.5	20.2	6.8	4.1	7.8
severe	1033.7	1040.4	6.9	29.2	6.1	3.2	7.0
severe	1012.5	838.3	5.1	12.9	6.3	4.0	7.4
severe	627.3	780.8	5.8	33.7	6.9	3.6	7.9

Supplementary Table S4: Metabolic data of *Nicotiana tabacum* under control conditions and different drought stress. The data of leaves (A), nectaries (B), and nectar (C) are presented in different tables.

(A) Leaves of *Nicotiana tabacum*

Treatment	Glucose [mM]	Fructose [mM]	Sucrose [mM]	Sum amino acids [mM]	Proline [mM]	Sum anions [mM]	Sum cations [mM]
control	12.7	26.4	18.6	19.0	0.3	164.6	332.1
control	23.5	25.4	10.2	17.7	0.8	115.7	294.9
control	18.4	18.6	13.7	18.1	0.8	169.5	265.2
control	6.8	13.1	4.7	22.3	1.2	164.0	198.4
mild	1.5	0.4	15.7	31.9	6.2	328.6	332.2
mild	9.6	4.4	23.4	34.7	3.2	250.7	400.8
mild	37.6	68.6	28.6	69.7	1.3	308.3	265.1
mild	2.6	1.6	33.6	32.3	6.1	199.8	333.3
severe	28.3	25.6	10.6	25.6	4.2	214.3	240.3
severe	103.1	71.7	9.1	31.6	12.8	311.6	302.7
severe	88.1	53.7	20.0	87.9	11.6	172.4	361.2
severe	70.7	52.1	16.3	104.3	5.4	150.9	404.2

(B) Nectaries of *Nicotiana tabacum*

Treatment	Glucose [mM]	Fructose [mM]	Sucrose [mM]	Sum amino acids [mM]	Proline [mM]	Sum anions [mM]	Sum cations [mM]
control	59.0	63.5	135.1	59.4	19.4	25.3	160.5
control	56.1	57.9	123.4	55.0	26.7	25.5	196.2
control	87.8	88.8	125.5	57.3	30.1	26.3	229.0
control	88.3	100.9	120.3	56.0	18.8	28.0	189.9
mild	24.1	55.7	47.8	64.6	28.0	28.7	186.4
mild	26.2	36.6	43.5	55.3	16.7	27.8	163.8
mild	94.0	118.7	103.9	62.1	24.5	28.6	218.3
mild	30.5	30.7	106.5	61.1	12.4	23.6	213.4
severe	14.1	20.4	144.5	82.3	46.4	28.9	226.5
severe	70.0	54.0	117.1	85.3	43.5	23.3	226.0
severe	61.3	33.8	108.3	77.2	45.0	29.4	267.1
severe	84.9	65.7	122.9	75.4	25.9	28.9	222.0

(C) Nectar of *Nicotiana tabacum*

Treatment	Glucose [mM]	Fructose [mM]	Sucrose [mM]	Sum amino acids [mM]	Proline [mM]	Sum anions [mM]	Sum cations [mM]
control	345.3	347.5	160.6	0.9	0.2	3.4	1.2
control	545.5	214.4	267.6	0.8	0.1	3.5	1.3
control	367.8	366.2	378.8	0.7	0.2	3.3	1.3
control	229.9	407.3	333.4	0.9	0.2	3.2	1.2
control	478.1	564.7	409.0	0.8	0.1	3.6	1.0
control	260.8	447.9	287.5	0.7	0.2	3.4	1.1
control	545.5	214.4	267.6	0.6	0.1	3.3	1.2
control	367.8	366.2	378.8	0.6	0.1	3.4	1.0
mild	517.8	532.7	281.7	1.2	0.2	3.7	1.3
mild	362.5	415.5	183.0	1.7	0.4	3.9	1.0
mild	275.8	330.0	270.3	2.2	0.5	4.0	1.4
mild	487.2	583.6	196.6	1.7	0.6	3.5	1.3
mild	447.7	478.5	218.6	1.2	0.4	4.4	1.0
mild	332.5	336.0	153.4	1.9	0.8	3.9	1.4
mild	509.6	766.9	212.2	2.4	0.4	4.9	1.2
mild	354.0	393.0	180.3	1.5	0.5	4.0	1.3
severe	607.1	995.7	265.9	2.1	0.8	6.1	2.4
severe	980.9	718.3	271.3	2.9	1.3	6.1	2.4
severe	457.3	1319.5	69.2	1.9	0.9	6.5	2.2
severe	928.1	1217.5	60.6	1.7	0.8	7.1	2.0
severe	1057.3	1069.6	80.9	3.0	1.8	7.6	2.0
severe	1077.1	1027.3	67.9	3.2	0.8	7.4	2.1
severe	1035.6	1122.8	80.1	2.6	1.2	7.1	2.2
severe	1058.5	628.7	90.1	2.4	1.1	6.8	2.4

Supplementary Table S5: Metabolic data of *Nicotiana sylvestris* under control conditions and different drought stress. The data of leaves (A), nectaries (B), and nectar (C) are presented in different tables.

(A) Leaves of *Nicotiana sylvestris*

Treatment	Glucose [mM]	Fructose [mM]	Sucrose [mM]	Sum amino acids [mM]	Proline [mM]	Sum anions [mM]	Sum cations [mM]
control	49.2	35.9	17.4	40.3	2.3	110.7	312.1
control	41.4	23.2	15.5	25.9	4.1	88.6	339.2
control	31.4	20.8	5.8	37.5	4.8	73.8	324.2
control	30.0	5.1	12.9	42.8	6.4	72.1	326.9
mild	40.0	23.4	19.3	34.1	4.2	109.8	329.1
mild	46.9	33.8	17.2	22.0	2.1	91.3	385.1
mild	82.8	29.9	16.8	31.6	4.2	150.8	374.2
mild	139.0	61.2	20.1	57.8	8.7	77.6	352.3
severe	32.8	32.6	26.6	31.0	4.2	274.3	415.6
severe	13.4	19.4	23.1	20.2	9.3	240.9	580.6
severe	71.1	34.0	5.1	26.8	2.6	196.4	295.0
severe	98.7	37.5	44.4	68.0	9.1	208.2	464.4

(B) Nectaries of *Nicotiana sylvestris*

Treatment	Glucose [mM]	Fructose [mM]	Sucrose [mM]	Sum amino acids [mM]	Proline [mM]	Sum anions [mM]	Sum cations [mM]
control	130.8	178.6	145.2	67.9	35.3	38.2	185.1
control	44.9	46.9	324.8	58.8	24.8	29.9	128.1
control	85.6	26.2	291.5	61.4	34.3	32.7	258.3
control	210.3	119.8	238.3	52.1	18.2	41.4	144.1
mild	153.5	56.3	215.8	92.3	63.3	42.8	222.9
mild	136.0	81.4	289.4	66.6	38.1	35.3	199.3
mild	79.7	65.0	179.3	52.3	25.2	22.5	226.8
mild	90.1	89.3	228.9	94.7	46.5	62.5	264.9
severe	209.4	248.5	87.2	186.1	24.4	112.9	180.7
severe	241.2	215.9	126.6	225.4	45.3	89.8	174.6
severe	171.7	149.5	483.3	210.3	43.3	85.5	241.1
severe	151.9	96.6	222.9	223.0	36.5	81.4	185.5

(C) Nectar of *Nicotiana sylvestris*

Treatment	Glucose [mM]	Fructose [mM]	Sucrose [mM]	Sum amino acids [mM]	Proline [mM]	Sum anions [mM]	Sum cations [mM]
control	200.2	174.0	556.7	1.1	0.3	5.2	3.6
control	297.9	286.2	657.3	1.5	0.3	4.6	3.8
control	294.2	338.2	331.5	1.1	0.4	5.7	3.0
control	150.1	179.5	522.2	0.9	0.2	5.0	3.7
control	215.8	394.9	355.2	0.9	0.2	5.7	3.3
control	156.6	362.9	402.0	0.9	0.2	5.8	3.6
control	215.1	388.4	371.4	0.5	0.1	5.2	3.1
control	179.3	270.3	399.6	0.8	0.3	5.6	3.2
mild	222.3	381.0	192.5	0.3	0.2	6.7	2.7
mild	433.0	649.0	341.0	0.4	0.2	7.1	3.0
mild	658.8	514.2	304.4	0.3	0.1	5.7	6.0
mild	760.7	491.5	218.2	0.3	0.2	6.8	6.8
mild	226.1	310.5	471.9	0.2	0.1	8.0	6.6
mild	285.1	581.7	305.9	0.6	0.1	7.6	3.0
mild	274.1	332.9	414.7	0.5	0.2	6.0	5.8
mild	673.2	496.6	337.7	0.3	0.2	6.8	5.7
severe	468.6	507.4	83.7	0.3	0.2	9.0	8.6
severe	893.0	783.8	87.7	0.4	0.3	10.8	9.6
severe	883.2	524.3	72.3	0.5	0.2	8.9	9.6
severe	492.0	558.8	93.9	0.4	0.1	9.8	9.1
severe	714.7	832.0	71.7	0.2	0.1	10.6	8.4
severe	1129.3	1003.3	77.2	0.2	0.0	8.6	9.0
severe	667.2	1184.0	71.7	0.2	0.0	11.0	10.7
severe	602.0	1021.0	79.3	0.2	0.0	9.7	11.1

Supplementary Table S6: Metabolic data of *Nicotiana otophora* under control conditions and different drought stress. The data of leaves (A), nectaries (B), and nectar (C) are presented in different tables.

(A) Leaves of *Nicotiana otophora*

Treatment	Glucose [mM]	Fructose [mM]	Sucrose [mM]	Sum amino acids [mM]	Proline [mM]	Sum anions [mM]	Sum cations [mM]
control	90.4	79.1	59.9	33.7	8.1	126.3	290.1
control	38.9	26.8	15.1	51.4	4.1	189.4	225.8
control	50.3	30.4	17.0	51.8	7.3	56.9	256.7
control	15.6	17.8	6.8	40.6	4.1	58.4	317.5
mild	45.8	43.2	30.7	15.1	10.5	63.2	375.1
mild	83.6	59.5	26.4	26.1	11.5	74.2	396.2
mild	135.1	117.3	10.1	90.2	4.1	137.6	493.8
mild	22.8	50.5	37.2	30.8	8.4	94.7	411.7
severe	12.7	11.5	11.8	98.4	5.6	80.8	531.7
severe	3.2	2.6	1.0	60.5	3.4	265.6	255.7
severe	1.1	1.9	2.2	77.7	6.5	168.6	809.3
severe	1.2	0.9	4.7	77.2	5.1	265.9	559.8

(B) Nectaries of *Nicotiana otophora*

Treatment	Glucose [mM]	Fructose [mM]	Sucrose [mM]	Sum amino acids [mM]	Proline [mM]	Sum anions [mM]	Sum cations [mM]
control	130.7	191.7	191.9	62.7	45.9	65.6	187.2
control	166.0	240.9	203.7	53.3	32.5	85.5	192.0
control	163.2	248.1	231.1	58.3	34.3	64.6	259.0
control	158.4	145.2	249.2	57.9	48.2	89.0	249.5
mild	160.8	106.6	215.3	71.5	72.1	52.8	203.6
mild	145.2	107.1	216.0	82.5	38.2	51.3	210.2
mild	208.5	230.7	233.5	71.9	45.4	57.6	239.1
mild	156.9	148.8	100.8	98.5	50.9	56.6	277.7
severe	164.7	220.3	179.0	130.8	95.9	34.7	311.3
severe	270.0	304.5	178.2	143.2	71.9	31.3	290.9
severe	178.1	171.8	204.9	146.7	48.5	33.8	169.2
severe	154.8	187.0	183.8	134.0	51.3	41.1	234.3

(C) Nectar of *Nicotiana otophora*

Treatment	Glucose [mM]	Fructose [mM]	Sucrose [mM]	Sum amino acids [mM]	Proline [mM]	Sum anions [mM]	Sum cations [mM]
control	356.3	347.0	165.4	0.5	0.3	0.9	4.9
control	397.2	398.0	129.2	0.7	0.2	1.0	4.9
control	356.7	315.4	200.2	1.4	0.2	1.1	4.7
control	581.0	424.2	167.4	1.4	0.3	1.3	4.9
control	315.1	277.2	139.4	1.7	0.4	1.2	5.0
control	454.2	478.8	68.1	2.5	0.0	1.1	5.3
control	387.8	374.3	205.5	2.7	1.8	0.6	4.6
control	341.2	412.9	138.7	1.4	0.5	1.1	5.1
mild	289.4	232.6	129.9	0.7	0.6	0.5	5.4
mild	230.6	218.0	116.8	0.6	0.1	0.5	5.6
mild	235.1	200.3	86.4	0.1	0.0	0.6	4.8
mild	294.8	181.0	103.3	0.2	0.2	0.6	4.6
mild	273.5	242.1	71.1	2.3	1.2	0.8	4.6
mild	226.1	184.2	136.6	2.4	1.0	0.6	4.1
mild	217.9	202.1	25.6	3.4	2.3	0.6	4.6
mild	210.4	212.1	151.1	2.2	0.7	0.8	5.4
severe	353.4	345.1	168.3	9.5	9.5	0.7	3.5
severe	369.2	317.7	100.7	1.0	1.0	0.8	3.3
severe	298.7	278.8	132.0	2.7	2.2	0.8	3.3
severe	289.4	232.6	129.9	1.5	1.4	0.6	3.3
severe	252.7	309.1	39.2	8.2	8.0	0.5	3.2
severe	373.6	371.2	136.0	9.8	9.3	0.9	3.4
severe	238.7	205.9	116.7	5.5	5.2	0.6	3.0
severe	225.9	197.8	72.5	7.6	7.3	0.7	3.1

Supplementary Table S7: Concentrations of various amino acids in leaves, nectaries and nectar of *Nicotiana* species under control conditions and different drought stress, *N. africana* (A), *N. tabacum* (B), *N. sylvestris* (C), and *N. otophora* (D). Different letters represent significant differences in leaves, nectaries, and nectar between control and drought conditions (Tukey's HSD; $p < 0.05$; leaf: n=3; nectary: n=4, nectar: n=8). Red color = The amino acid concentration in plants under severe drought stress is significantly higher than in plants under control conditions. Blue color: The amino acid concentration in plants under severe drought stress is significantly lower than in plants under control conditions.

(A) *Nicotiana africana*

	**Leaves under different treatment [mM]**			**Nectaries under different treatment [mM]**			**Nectar under different treatment [mM]**		
	**control**	**mild**	**severe**	**control**	**mild**	**severe**	**control**	**mild**	**severe**
Ala	0.2 ± 0.0^{a}	0.3 ± 0.1^{a}	1.6 ± 1.0^{b}	3.0 ± 1.4^{a}	3.4 ± 1.9^{a}	12.2 ± 7.0^{b}	0.6 ± 0.5^{a}	2.0 ± 1.3^{b}	3.1 ± 1.1^{b}
Arg	0.03 ± 0.03^{a}	0.5 ± 0.7^{a}	0.4 ± 0.1^{a}	0.4 ± 0.2^{a}	0.3 ± 0.1^{a}	0.3 ± 0.1^{a}	0.2 ± 0.1^{a}	0.3 ± 0.1^{a}	0.3 ± 0.2^{a}
Asn	0.9 ± 0.2^{a}	2.0 ± 1.4^{a}	0.6 ± 0.1^{a}	17.3 ± 10.6^{a}	6.1 ± 1.0^{a}	8.8 ± 4.2^{a}	0.3 ± 0.2^{a}	2.1 ± 1.1^{a}	15.1 ± 8.3^{b}
Asp	0.3 ± 0.2^{a}	2.1 ± 1.1^{a}	15.1 ± 8.3^{b}	1.2 ± 0.2^{a}	1.5 ± 0.3^{a}	1.6 ± 0.4^{a}	0.9 ± 0.2^{a}	2.0 ± 1.4^{a}	0.6 ± 0.1^{a}
Gln	1.4 ± 0.3^{ab}	2.9 ± 1.5^{a}	0.9 ± 0.1^{b}	19.1 ± 8.1^{a}	27.6 ± 6.0^{a}	30.6 ± 5.2^{a}	0.5 ± 0.4^{a}	4.6 ± 2.7^{ab}	10.0 ± 6.5^{b}
Glu	0.5 ± 0.4^{a}	4.6 ± 2.7^{ab}	10.0 ± 6.5^{b}	3.7 ± 1.4^{a}	4.6 ± 1.2^{a}	7.1 ± 1.0^{b}	1.4 ± 0.3^{ab}	2.9 ± 1.5^{a}	0.9 ± 0.1^{b}
Gly	0.1 ± 0.0^{a}	0.2 ± 0.1^{a}	0.1 ± 0.0^{a}	0.4 ± 0.0^{a}	0.5 ± 0.1^{a}	0.6 ± 0.2^{a}	0.1 ± 0.0^{a}	0.2 ± 0.1^{b}	0.2 ± 0.1^{b}
His	0.02 ± 0.01^{a}	0.6 ± 0.8^{a}	1.0 ± 0.8^{a}	1.3 ± 0.3^{a}	2.0 ± 0.3^{ab}	3.1 ± 1.6^{b}	0.02 ± 0.01^{a}	0.6 ± 0.8^{a}	1.0 ± 0.8^{a}
Ile	0.1 ± 0.0^{a}	0.6 ± 0.5^{b}	0.2 ± 0.0^{ab}	0.5 ± 0.2^{a}	0.4 ± 0.1^{a}	0.4 ± 0.2^{a}	0.1 ± 0.1^{a}	0.3 ± 0.2^{a}	0.3 ± 0.2^{a}
Leu	0.1 ± 0.0^{a}	0.5 ± 0.3^{a}	0.2 ± 0.1^{a}	0.9 ± 0.3^{a}	1.4 ± 0.1^{b}	1.2 ± 0.1^{ab}	0.5 ± 0.1^{a}	0.5 ± 0.1^{a}	0.5 ± 0.1^{a}
Lys	0.3 ± 0.0^{a}	0.3 ± 0.0^{a}	0.2 ± 0.1^{a}	0.5 ± 0.0a	0.7 ± 0.2^{a}	0.5 ± 0.3^{a}	0.03 ± 0.01^{a}	0.04 ± 0.02^{a}	0.04 ± 0.02^{a}
Met	0.05 ± 0.01^{a}	0.3 ± 0.2^{a}	0.8 ± 0.3^{b}	0.7 ± 0.2^{a}	0.7 ± 0.1^{a}	0.9 ± 0.5^{a}	0.01 ± 0.00^{a}	0.02 ± 0.02^{a}	0.02 ± 0.01^{a}
Phe	0.2 ± 0.2^{a}	1.9 ± 1.4^{a}	1.7 ± 0.5^{a}	0.8 ± 0.6^{a}	2.3 ± 0.6^{b}	1.4 ± 0.4^{a}	0.7 ± 0.1^{a}	0.9 ± 0.3^{a}	1.0 ± 0.2^{a}
Pro	1.0 ± 0.5^{a}	3.7 ± 1.7^{b}	6.4 ± 1.1^{c}	15.0 ± 7.1^{a}	55.6 ± 20.3^{b}	70.1 ± 15.1^{b}	4.4 ± 0.7^{a}	5.6 ± 0.4^{b}	6.3 ± 0.4^{c}
Ser	0.2 ± 0.1^{a}	0.3 ± 0.1^{a}	0.4 ± 0.2^{a}	2.8 ± 1.0^{a}	4.5 ± 0.7^{b}	5.5 ± 0.7^{b}	0.2 ± 0.1^{a}	0.3 ± 0.1^{a}	0.4 ± 0.2^{a}
Thr	0.1 ± 0.0^{a}	0.3 ± 0.2^{ab}	0.5 ± 0.1^{b}	1.4 ± 0.4^{ab}	2.0 ± 0.3^{a}	1.3 ± 0.4^{b}	0.3 ± 0.1^{a}	0.5 ± 0.2^{b}	0.5 ± 0.1^{b}
Trp	0.1 ± 0.1^{a}	1.0 ± 0.6^{b}	0.7 ± 0.0^{ab}	1.3 ± 0.4^{a}	1.2 ± 0.2^{a}	0.8 ± 0.4^{a}	0.01 ± 0.01^{a}	0.02 ± 0.02^{a}	0.02 ± 0.02^{a}
Tyr	0.03 ± 0.01^{a}	0.1 ± 0.1^{a}	0.1 ± 0.0^{a}	0.9 ± 0.6^{a}	0.5 ± 0.3^{a}	0.5 ± 0.1^{a}	0.1 ± 0.0^{a}	0.1 ± 0.0^{a}	0.1 ± 0.0^{a}
Val	0.1 ± 0.1^{a}	1.4 ± 1.0^{b}	1.1 ± 0.2^{ab}	0.8 ± 0.2^{a}	1.4 ± 0.3^{b}	1.1 ± 0.2^{ab}	0.3 ± 0.1^{a}	0.5 ± 0.2^{b}	0.6 ± 0.2^{b}

(B) *Nicotiana tabacum*

	Leaves under different treatment [mM]			Nectaries under different treatment [mM]			Nectar under different treatment [mM]		
	**control**	**mild**	**severe**	**control**	**mild**	**severe**	**control**	**mild**	**severe**
Ala	0.8 ± 0.1[a]	1.2 ± 0.3[a]	1.4 ± 0.9[a]	0.4 ± 0.1[a]	0.5 ± 0.1[a]	0.5 ± 0.2[a]	0.01 ± 0.01[a]	0.03 ± 0.01[a]	0.03 ± 0.02[a]
Arg	0.2 ± 0.1[a]	0.9 ± 0.5[a]	2.6 ± 2.1[a]	0.1 ± 0.0[a]	0.1 ± 0.0[a]	0.1 ± 0.1[a]	0.00 ± 0.00[a]	0.01 ± 0.01[ab]	0.01 ± 0.01[b]
Asn	1.3 ± 0.4[a]	2.1 ± 1.3[a]	4.3 ± 3.0[a]	11.9 ± 6.1[a]	14.1 ± 3.8[a]	10.2 ± 3.2[a]	0.2 ± 0.1[a]	0.6 ± 0.2[b]	0.6 ± 0.2[b]
Asp	4.3 ± 0.1[a]	7.3 ± 2.5[a]	3.8 ± 1.8[a]	2.0 ± 0.4[a]	2.1 ± 0.4[a]	2.2 ± 0.4[a]	0.1 ± 0.0[a]	0.1 ± 0.1[a]	0.1 ± 0.1[a]
Gln	1.4 ± 1.0[a]	4.9 ± 3.4[a]	9.5 ± 7.3[a]	8.2 ± 1.5[a]	11.5 ± 3.0[ab]	14.7 ± 4.2[b]	0.1 ± 0.0[a]	0.3 ± 0.2[ab]	0.4 ± 0.2[b]
Glu	6.9 ± 0.2[a]	11.9 ± 5.3[a]	7.6 ± 5.5[a]	3.7 ± 1.0[a]	4.3 ± 1.2[a]	3.9 ± 0.7[a]	0.0 ± 0.0[a]	0.1 ± 0.0[ab]	0.1 ± 0.0[b]
Gly	0.1 ± 0.0[a]	0.5 ± 0.2[a]	1.9 ± 2.1[a]	0.2 ± 0.0[a]	0.2 ± 0.1[a]	0.3 ± 0.1[a]	0.03 ± 0.01[a]	0.04 ± 0.01[a]	0.04 ± 0.01[a]
His	0.2 ± 0.1[a]	0.6 ± 0.4[a]	3.8 ± 3.9[a]	1.1 ± 0.5[a]	1.5 ± 0.3[a]	1.6 ± 0.3[a]	0.00 ± 0.00[a]	0.00 ± 0.00[ab]	0.01 ± 0.00[b]
Ile	0.2 ± 0.1[a]	0.4 ± 0.2[a]	2.1 ± 2.3[a]	0.2 ± 0.1[a]	0.3 ± 0.1[a]	0.3 ± 0.1[a]	0.01 ± 0.00[a]	0.01 ± 0.00[a]	0.01 ± 0.00[a]
Leu	0.1 ± 0.0[a]	0.5 ± 0.3[a]	0.8 ± 0.6[a]	0.6 ± 0.1[a]	0.7 ± 0.2[a]	0.7 ± 0.1[a]	0.01 ± 0.00[a]	0.01 ± 0.00[a]	0.01 ± 0.00[a]
Lys	0.1 ± 0.0[a]	0.3 ± 0.2[a]	2.5 ± 3.5[a]	0.1 ± 0.0[a]	0.2 ± 0.1[a]	0.2 ± 0.1[a]	0.00 ± 0.00[a]	0.01 ± 0.01[a]	0.01 ± 0.01[a]
Met	0.3 ± 0.2[a]	0.2 ± 0.1[a]	0.7 ± 0.8[a]	0.2 ± 0.0[a]	0.3 ± 0.1[a]	0.3 ± 0.1[a]	0.00 ± 0.01[a]	0.00 ± 0.00[a]	0.00 ± 0.00[a]
Phe	0.2 ± 0.1[a]	3.1 ± 3.1[a]	5.2 ± 7.0[a]	0.4 ± 0.0[a]	0.4 ± 0.2[a]	0.4 ± 0.1[a]	0.00 ± 0.00[a]	0.01 ± 0.00[ab]	0.01 ± 0.00[b]
Pro	1.5 ± 1.5[a]	3.5 ± 2.4[a]	7.1 ± 4.0[a]	23.8 ± 5.6[a]	20.4 ± 7.1[a]	40.2 ± 9.6[b]	0.2 ± 0.0[a]	0.5 ± 0.2[b]	1.1 ± 0.4[c]
Ser	0.7 ± 0.1[a]	1.6 ± 1.1[a]	2.6 ± 2.4[a]	2.7 ± 0.5[a]	2.7 ± 0.6[a]	2.8 ± 0.4[a]	0.02 ± 0.01[a]	0.04 ± 0.01[b]	0.04 ± 0.02[b]
Thr	0.5 ± 0.0[a]	1.6 ± 1.4[a]	3.5 ± 2.6[a]	0.6 ± 0.1[a]	0.6 ± 0.1[a]	0.7 ± 0.1[a]	0.00 ± 0.00[a]	0.01 ± 0.00[a]	0.01 ± 0.00[a]
Trp	0.6 ± 0.3[a]	2.1 ± 1.8a	3.9 ± 2.2[a]	0.2 ± 0.1[a]	0.2 ± 0.1[a]	0.1 ± 0.1[a]	0.00 ± 0.00[a]	0.00 ± 0.00[b]	0.00 ± 0.00[ab]
Tyr	0.1 ± 0.0[a]	0.8 ± 0.5[a]	2.9 ± 2.4[a]	0.2 ± 0.1[a]	0.2 ± 0.1[a]	0.2 ± 0.1[a]	0.00 ± 0.00[a]	0.01 ± 0.00[b]	0.01 ± 0.00[b]
Val	0.4 ± 0.0[a]	1.9 ± 0.9[a]	6.5 ± 6.4[a]	0.4 ± 0.0[a]	0.5 ± 0.1[a]	0.4 ± 0.1[a]	0.02 ± 0.00[a]	0.03 ± 0.02[a]	0.03 ± 0.02[a]

(C) *Nicotiana sylvestris*

	**Leaves under different treatment [mM]**			**Nectaries under different treatment [mM]**			**Nectar under different treatment [mM]**		
	**control**	**mild**	**severe**	**control**	**mild**	**severe**	**control**	**mild**	**severe**
Ala	0.9 ± 0.2[a]	0.9 ± 0.1[a]	1.8 ± 1.6[a]	0.8 ± 0.4[a]	0.6 ± 0.2[a]	1.8 ± 0.4[b]	0.03 ± 0.03[a]	0.01 ± 0.00[b]	0.01 ± 0.00[b]
Arg	0.6 ± 0.5[a]	0.7 ± 0.4[a]	1.4 ± 1.2[a]	0.2 ± 0.1[a]	0.3 ± 0.1[a]	1.1 ± 0.7[b]	0.00 ± 0.00[a]	0.00 ± 0.00[a]	0.00 ± 0.00[a]
Asn	2.4 ± 1.0[a]	1.2 ± 0.4[a]	2.6 ± 0.9[a]	4.4 ± 1.3[a]	5.2 ± 2.8[a]	52.1 ± 9.5[b]	0.2 ± 0.1[a]	0.04 ± 0.03[b]	0.02 ± 0.01[b]
Asp	5.9 ± 1.7[a]	3.6 ± 0.9[a]	4.5 ± 1.6[a]	3.0 ± 0.9[a]	3.4 ± 0.8[a]	19.6 ± 2.2[b]	0.1 ± 0.0[a]	0.01 ± 0.00[b]	0.01 ± 0.00[b]
Gln	2.6 ± 0.4[a]	1.7 ± 0.6[a]	3.2 ± 2.3[a]	7.8 ± 1.1[a]	6.3 ± 2.7[a]	40.8 ± 8.7[b]	0.14 ± 0.07[a]	0.05 ± 0.04[b]	0.03 ± 0.01[b]
Glu	8.3 ± 1.6[a]	7.2 ± 1.1[a]	6.5 ± 0.5[a]	3.4 ± 2.8[a]	4.9 ± 0.7[a]	18.0 ± 1.6[b]	0.1 ± 0.1[a]	0.01 ± 0.00[b]	0.01 ± 0.00[b]
Gly	0.3 ± 0.1[a]	0.4 ± 0.0[a]	0.4 ± 0.0[a]	0.2 ± 0.0[a]	0.3 ± 0.1[a]	1.5 ± 0.6[b]	0.02 ± 0.02[a]	0.01 ± 0.01[a]	0.03 ± 0.00[a]
His	0.5 ± 0.4[a]	0.3 ± 0.1[a]	0.6 ± 0.4[a]	0.5 ± 0.4[a]	2.1 ± 1.8[ab]	4.9 ± 2.8[b]	0.04 ± 0.06[a]	0.02 ± 0.03[a]	0.00 ± 0.00[a]
Ile	0.9 ± 0.8[a]	1.1 ± 0.3[a]	1.3 ± 1.2[a]	0.2 ± 0.1[a]	0.6 ± 0.5[ab]	2.0 ± 1.6[b]	0.01 ± 0.00[a]	0.00 ± 0.00[a]	0.00 ± 0.00[a]
Leu	0.7 ± 0.6[a]	0.7 ± 0.2[a]	0.8 ± 0.8[a]	0.9 ± 0.1[a]	1.2 ± 0.2[a]	2.0 ± 0.5[b]	0.01 ± 0.00[a]	0.01 ± 0.00[b]	0.00 ± 0.00[b]
Lys	0.4 ± 0.2[a]	0.4 ± 0.2[a]	0.6 ± 0.4[a]	0.3 ± 0.0[a]	0.2 ± 0.0[a]	1.2 ± 0.7[b]	0.00 ± 0.00[a]	0.00 ± 0.00[b]	0.00 ± 0.00[b]
Met	0.5 ± 0.0[a]	0.6 ± 0.1[a]	0.9 ± 0.5[a]	1.4 ± 0.9[a]	0.9 ± 0.6[a]	0.3 ± 0.2[a]	0.01 ± 0.01[a]	0.00 ± 0.01[b]	0.00 ± 0.00[b]
Phe	1.0 ± 0.6[a]	1.0 ± 0.3[a]	2.9 ± 4.0[a]	1.0 ± 0.2[a]	0.7 ± 0.2[a]	3.5 ±1.2[b]	0.01 ± 0.01[a]	0.00 ± 0.00[b]	0.01 ± 0.00[b]
Pro	4.3 ± 2.1[a]	3.5 ± 1.2[a]	5.3 ± 3.4[a]	28.2 ± 8.1[a]	43.3 ± 16.0[a]	37.4 ± 9.4[a]	0.2 ± 0.1a	0.2 ± 0.1ab	0.1 ± 0.1b
Ser	2.2 ± 0.2[a]	1.7 ± 0.3[a]	2.3 ± 0.7[a]	5.4 ± 1.0[a]	3.8 ± 0.6[a]	14.0 ± 4.3[b]	0.06 ± 0.03[a]	0.01 ± 0.01[b]	0.02 ± 0.01[b]
Thr	1.1 ± 0.3[a]	0.9 ± 0.3[a]	1.6 ± 0.7[a]	1.3 ± 0.3[a]	1.1 ± 0.2[a]	4.3 ± 0.5[b]	0.01 ± 0.00[a]	0.01 ± 0.00[b]	0.00 ± 0.00[b]
Trp	0.5 ± 0.3[ab]	1.2 ± 0.2[a]	2.3 ± 1.4[b]	0.4 ± 0.3[a]	0.7 ± 0.1[ab]	2.2 ± 1.4[b]	0.00 ± 0.00[a]	0.00 ± 0.00[a]	0.00 ± 0.00[a]
Tyr	0.4 ± 0.3[a]	0.5 ± 0.2[a]	0.8 ± 0.7[a]	0.2 ± 0.0[a]	0.2 ± 0.1[a]	1.0 ± 0.2[b]	0.01 ± 0.00[a]	0.00 ± 0.00[b]	0.00 ± 0.00[b]
Val	2.0 ± 0.8[a]	1.7 ± 0.6[a]	2.2 ± 2.3[a]	0.4 ± 0.0[a]	0.6 ± 0.3[a]	3.6 ± 1.8[b]	0.01 ± 0.00[ab]	0.01 ± 0.00[a]	0.01 ± 0.00[b]

(D) *Nicotiana otophora*

	Leaves under different treatment [mM]			Nectaries under different treatment [mM]			Nectar under different treatment [mM]		
	**control**	**mild**	**severe**	**control**	**mild**	**severe**	**control**	**mild**	**severe**
Ala	1.7 ± 0.4[a]	0.5 ± 0.3[b]	2.8 ± 0.2[c]	0.7 ± 0.2[a]	1.3 ± 0.5[b]	1.1 ± 0.2[ab]	0.03 ± 0.02[a]	0.01 ± 0.01[ab]	0.00 ± 0.01[b]
Arg	0.2 ± 0.2[a]	0.2 ± 0.1[a]	0.9 ± 0.2[b]	0.1 ± 0.0[ab]	0.1 ± 0.0[a]	0.1 ± 0.0[b]	0.01 ± 0.01[a]	0.01 ± 0.01[a]	0.00 ± 0.00[a]
Asn	11.8 ± 9.2[a]	1.9 ± 1.7[a]	23.4 ± 5.1[b]	1.7 ± 0.8[a]	3.6 ± 1.3[a]	31.1 ± 15.7[b]	0.4 ± 0.4[a]	0.3 ± 0.2[a]	0.1 ± 0.1[a]
Asp	8.0 ± 0.7[a]	2.7 ± 2.2[b]	4.6 ± 1.5[b]	1.5 ± 0.3[a]	1.9 ± 0.4[a]	2.0 ± 0.5[a]	0.1 ± 0.1[a]	0.1 ± 0.1[b]	0.02 ± 0.02[b]
Gln	6.9 ± 1.9[a]	1.6 ± 1.1[a]	22.6 ± 8.3[b]	4.3 ± 2.3[a]	10.6 ± 4.3[a]	24.1 ± 8.7[b]	0.1 ± 0.1[a]	0.2 ± 0.3[a]	0.1 ± 0.1[a]
Glu	5.4 ± 7.6[a]	3.1 ± 1.7[a]	5.5 ± 1.0[a]	4.6 ± 1.5[a]	5.7 ± 0.9[a]	3.9 ± 0.6[a]	0.2 ± 0.2[a]	0.04 ± 0.03[ab]	0.01 ± 0.02[b]
Gly	0.5 ± 0.5[a]	0.6 ± 0.2[a]	0.5 ± 0.1[a]	0.1 ± 0.1[a]	0.2 ± 0.0[ab]	0.3 ± 0.1[b]	0.03 ± 0.01[a]	0.02 ± 0.02[a]	0.00 ± 0.00[b]
His	0.3 ± 0.1[a]	0.1 ± 0.0[a]	0.8 ± 0,4[b]	0.1 ± 0.1[a]	0.8 ± 0.2[a]	2.7 ± 1.4[b]	0.01 ± 0.01[a]	0.00 ± 0.00[a]	0.00 ± 0.00[a]
Ile	0.1 ± 0.1[a]	0.3 ± 0.1[a]	0.2 ± 0.1[a]	0.1 ± 0.0[a]	0.1 ± 0.0[b]	0.1 ± 0.0[a]	0.01 ± 0.00[a]	0.00 ± 0.00[ab]	0.00 ± 0.00[b]
Leu	0.2 ± 0.0[a]	0.3 ± 0.1[a]	0.4 ± 0.1[a]	0.6 ± 0.1[a]	0.6 ± 0.1[a]	0.6 ± 0.1[a]	0.01 ± 0.01[a]	0.01 ± 0.01[a]	0.00 ± 0.00[a]
Lys	0.3 ± 0.2[a]	0.6 ± 0.6[a]	0.8 ± 0.3[a]	0.1 ± 0.0[a]	0.1 ± 0.0[ab]	0.1 ± 0.1[b]	0.03 ± 0.03[a]	0.02 ± 0.02[ab]	0.00 ± 0.00[b]
Met	1.2 ± 0.7[a]	0.6 ± 0.6[a]	1.3 ± 0.2[a]	0.2 ± 0.1[a]	0.4 ± 0.2[ab]	0.6 ± 0.1[b]	0.00 ± 0.00[a]	0.01 ± 0.01[a]	0.00 ± 0.01[a]
Phe	0.5 ± 0.3[a]	0.4 ± 0.1[a]	1.3 ± 0.1[b]	0.6 ± 0.2[a]	0.8 ± 0.4[a]	1.1 ± 0.4[a]	0.03 ± 0.02[a]	0.02 ± 0.03[a]	0.00 ± 0.00[a]
Pro	6.5 ± 2.1[a]	7.7 ± 3.3[a]	4.7 ± 1.2[a]	40.2 ± 8.0[a]	51.7 ± 14.6[a]	66.9 ± 22.0[a]	0.5 ± 0.6[a]	0.8 ± 0.8[a]	5.5 ± 3.5[b]
Ser	2.8 ± 0.3[a]	1.5 ± 0.3[b]	1.4 ± 0.3[b]	2.2 ± 0.7[a]	2.8 ± 0.5[a]	2.3 ± 0.3[a]	0.05 ± 0.03[a]	0.03 ± 0.03[ab]	0.01 ± 0.01[b]
Thr	0.8 ± 0.6[a]	0.5 ± 0.2[a]	2.5 ± 0.1[b]	0.2 ± 0.1[a]	0.3 ± 0.1[a]	0.7 ± 0.2[b]	0.01 ± 0.01[a]	0.01 ± 0.01[a]	0.00 ± 0.00[a]
Trp	0.3 ± 0.4[a]	0.4 ± 0.2[a]	1.2 ± 0.3[b]	0.04 ± 0.01[a]	0.03 ± 0.02[a]	0.07 ± 0.04[a]	0.02 ± 0.03[a]	0.00 ± 0.00[a]	0.00 ± 0.00[a]
Tyr	0.2 ± 0.1[a]	0.2 ± 0.1[ab]	0.6 ± 0.1[b]	0.2 ± 0.1[a]	0.1 ± 0.0[a]	0.1 ± 0.0[a]	0.00 ± 0.00[a]	0.00 ± 0.00[ab]	0.00 ± 0.00[b]
Val	0.3 ± 0.1[a]	0.8 ± 0.3[b]	1.7 ± 0.2[c]	0.4 ± 0.1[a]	0.4 ± 0.1[a]	0.7 ± 0.2[b]	0.04 ± 0.02[a]	0.02 ± 0.01[b]	0.00 ± 0.00[b]

Supplementary Table S8: Concentrations of various inorganic ions in leaves, nectaries and nectar of *Nicotiana* species under control conditions and different drought stress, *N. africana* (A), *N. tabacum* (B), *N. sylvestris* (C), and *N. otophora* (D). Different letters represent significant differences in leaves, nectaries, and nectar between control and drought conditions (Tukey's HSD; $p < 0.05$; leaf: n=3; nectary: n=4, nectar: n=8). Red color = The inorganic ion concentration in plants under severe drought stress is significantly higher than in plants under control conditions. Blue color: The inorganic ion concentration in plants under severe drought stress is significantly lower than in plants under control conditions.

(A) *Nicotiana africana*

	**Leaves under different treatment [mM]**			**Nectaries under different treatment [mM]**			**Nectar under different treatment [mM]**		
	**control**	**mild**	**severe**	**control**	**mild**	**severe**	**control**	**mild**	**severe**
K^+	282.9 ± 108.8^{a}	427.8 ± 221.8^{ab}	661.5 ± 56.7^{b}	81.8 ± 9.9^{a}	151.5 ± 19.7^{b}	182.8 ± 15.6^{c}	3.0 ± 0.2^{a}	4.5 ± 0.7^{b}	6.4 ± 0.3^{c}
Na^+	20.6 ± 33.7^{a}	65.5 ± 55.0^{a}	209.6 ± 7.9^{b}	6.4 ± 2.0^{a}	10.1 ± 4.3^{ab}	12.1 ± 1.3^{b}	0.01 ± 0.01^{a}	0.01 ± 0.00^{a}	0.02 ± 0.03^{a}
Mg^{2+}	10.7 ± 5.0^{a}	22.3 ± 3.7^{b}	42.3 ± 4.6^{c}	5.9 ± 3.1^{a}	19.6 ± 4.0^{b}	34.3 ± 2.5^{c}	0.01 ± 0.01^{a}	0.02 ± 0.01^{ab}	0.05 ± 0.04^{b}
Ca^{2+}	18.7 ± 4.8^{a}	43.0 ±3.3^{b}	153.2 ± 7.3^{c}	43.8 ± 5.1^{a}	38.7 ± 17.3^{ab}	24.4 ± 3.0^{b}	0.3 ± 0.1^{a}	0.4 ± 0.2^{a}	0.4 ± 0.2^{a}
NH_4^+	4.7 ± 0.7^{a}	7.8 ± 1.3^{a}	19.3 ± 2.8^{b}	3.6 ± 2.0^{a}	6.4 ± 5.0^{a}	6.6 ± 4.7^{a}	0.1 ± 0.0^{a}	0.2 ± 0.2^{ab}	0.4 ± 0.2^{b}
Cl^-	138.4 ± 16.1^{a}	119.5 ± 45.9^{a}	260.3 ± 53.4^{b}	3.3 ± 0.3^{a}	10.2 ± 5.0^{b}	12.4 ± 3.0^{b}	1.5 ± 0.2^{a}	1.5 ± 0.3^{a}	3.1 ± 0.4^{b}
PO_4^{3-}	32.0 ± 8.9^{a}	16.0 ± 6.6^{a}	40.7 ± 35.9^{a}	1.0 ± 0.8^{a}	4.8 ± 4.7^{a}	9.9 ± 7.5^{a}	0.2 ± 0.1^{a}	0.2 ± 0.1^{a}	0.4 ± 0.2^{b}
SO_4^{2-}	14.5 ± 2.0^{a}	116.9 ± 83.9^{a}	89.9 ± 64.2^{a}	6.9 ± 2.0^{a}	35.9 ± 19.6^{b}	42.7 ± 10.3^{b}	0.1 ± 0.1^{a}	0.1 ± 0.0^{a}	0.1 ± 0.0^{a}

(B) *Nicotiana tabacum*

	**Leaves under different treatment [mM]**			**Nectaries under different treatment [mM]**			**Nectar under different treatment [mM]**		
	**control**	**mild**	**severe**	**control**	**mild**	**severe**	**control**	**mild**	**severe**
K^+	222.7 ± 38.1^{a}	293.1 ± 48.9^{a}	305.2 ± 43.1^{a}	121.9 ± 28.3^{a}	119.7 ± 15.9^{a}	130.6 ± 21.8^{a}	0.9 ± 0.1^{a}	0.8 ± 0.2^{a}	1.9 ± 0.2^{b}
Na^+	5.8 ± 5.4^{a}	13.0 ± 12.4^{a}	8.8 ± 0.7^{a}	9.0 ± 1.6^{a}	9.2 ± 3.6^{a}	13.4 ± 1.7^{a}	0.04 ± 0.02^{a}	0.03 ± 0.01^{a}	0.04 ± 0.02^{a}
Mg^{2+}	19.8 ± 4.8^{a}	20.2 ± 5.3^{a}	35.6 ± 7.0^{b}	42.6 ± 6.7^{a}	44.5 ± 8.5^{a}	59.5 ± 6.5^{b}	0.1 ± 0.0^{a}	0.1 ± 0.0^{a}	0.1 ± 0.0^{b}
Ca^{2+}	2.1 ± 1.4 ^{a}	2.5 ± 0.7^{b}	1.8 ± 1.0^{b}	15.1 ± 4.9^{a}	14.4 ± 3.1^{a}	21.8 ± 1.9^{b}	0.1 ± 0.0^{a}	0.1 ± 0.0^{a}	0.1 ± 0.0^{a}
NH_4^+	2.2 ± 0.5^{a}	3.5 ± 1.6^{a}	4.3 ± 4.4^{a}	5.3 ± 1.6^{a}	7.6 ± 2.7^{ab}	10.0 ± 0.7^{b}	0.1 ± 0.0^{a}	0.3 ± 0.2^{b}	0.2 ± 0.0^{ab}
Cl^-	102.8 ± 17.5^{a}	131.9 ± 17.9^{a}	145.4 ± 35.3^{a}	8.2 ± 1.7^{a}	15.2 ± 4.0^{b}	11.4 ± 2.6^{ab}	2.7 ± 0.2^{a}	3.4 ± 0.4^{b}	5.8 ± 0.5^{c}
PO_4^{3-}	47.0 ± 11.3^{a}	52.9 ± 36.6^{a}	23.3 ± 7.4^{a}	2.6 ± 2.0^{a}	0.00 ± 0.00^{b}	0.4 ± 0.8^{b}	0.5 ± 0.1^{a}	0.4 ± 0.3^{a}	0.8 ± 0.2^{b}
SO_4^{2-}	16.0 ± 9.1^{a}	14.3 ± 18.0^{a}	10.2 ± 4.4^{a}	15.4 ± 3.1^{a}	12.0 ± 1.9^{a}	15.8 ± 3.6^{a}	0.2 ± 0.1ab	0.2 ± 0.1a	0.2 ± 0.0b

(C) *Nicotiana sylvestris*

	Leaves under different treatment [mM]			Nectaries under different treatment [mM]			Nectar under different treatment [mM]		
	**control**	**mild**	**severe**	**control**	**mild**	**severe**	**control**	**mild**	**severe**
K^+	261.4 ± 10.6[a]	285.1 ± 7.7[a]	314.4 ± 79.1[a]	110.5 ± 41.7[a]	97.1 ± 6.0[a]	125.3 ± 21.9[a]	1.8 ± 0.5[a]	3.6 ± 1.3a	6.8 ± 1.0[c]
Na^+	33.2 ± 8.3[a]	42.2 ± 14.8[a]	34.2 ± 31.9[a]	10.4 ± 2.5[a]	24.9 ± 10.3[b]	12.2 ± 1.8[ab]	0.1 ± 0.0[ab]	0.04 ± 0.01[a]	1.0 ± 0.0[b]
Mg^{2+}	24.7 ± 1.0[a]	31.4 ± 2.5[a]	31.3 ± 2.5[a]	32.0 ± 14.8[ab]	47.7 ± 5.6[a]	23.2 ± 3.2[b]	0.1 ± 0.0[a]	0.1 ± 0.1[a]	0.3 ± 0.2[b]
Ca^{2+}	2.4 ± 0.6[a]	2.8 ± 0.5[a]	4.5 ± 3.1[a]	18.6 ± 2.5[a]	51.6 ± 23.3[b]	27.9 ± 5.6[ab]	0.7 ± 0.2[a]	0.2 ± 0.2[b]	1.1 ± 0.3[c]
NH_4^+	7.2 ± 1.6[a]	7.7 ± 2.2[a]	5.9 ± 1.8[a]	7.3 ± 1.9[a]	7.1 ± 0.8[a]	6.9 ± 1.6[a]	0.8 ± 0.1[a]	1.0 ± 0.3[ab]	1.3 ± 0.3[b]
Cl^-	48.0 ± 6.8[a]	57.3 ± 22.2[a]	86.5 ± 39.5[a]	8.3 ± 2.1[a]	34.4 ± 48.4[a]	26.3 ± 9.7[a]	4.7 ± 0.5[a]	6.1 ± 0.8[b]	8.5 ± 0.9[c]
PO_4^{3-}	2.6 ± 0.0[a]	1.3 ± 1.1[a]	4.6 ± 4.0[a]	13.0 ± 9.1[a]	10.3 ± 12.0[a]	11.8 ± 3.5[a]	0.2 ± 0.1[a]	0.2 ± 0.1[a]	0.2 ± 0.1[a]
SO_4^{2-}	27.3 ± 2.3[a]	47.6 ± 17.2[ab]	72.7 ± 33.5[b]	14.3 ± 9.3[a]	8.7 ± 1.9[a]	41.7 ± 6.2[b]	0.4 ± 0.2[a]	0.5 ± 0.1[a]	0.6 ± 0.2[a]

(D) *Nicotiana otophora*

	Leaves under different treatment [mM]			Nectaries under different treatment [mM]			Nectar under different treatment [mM]		
	**control**	**mild**	**severe**	**control**	**mild**	**severe**	**control**	**mild**	**severe**
K^+	249.0 ± 16.1[a]	338.0 ± 45.5[a]	551.1 ± 108.0[b]	178.0 ± 37.6[a]	179.2 ± 22.2[a]	159.8 ± 35.3[a]	3.9 ± 0.3[a]	3.9 ± 0.5[a]	2.5 ± 0.2[b]
Na^+	5.7 ± 2.7[ab]	17.1 ± 9.6[a]	5.0 ± 1.5[b]	6.5 ± 0.8[a]	6.2 ± 2.7[a]	37.1 ± 51.3[a]	0.1 ± 0.0[a]	0.1 ± 0.0[a]	0.1 ± 0.0[a]
Mg^{2+}	57.8 ± 24.2[a]	61.5 ± 4.0[a]	39.1 ± 21.1[a]	16.3 ± 1.9[a]	10.5 ± 3.4[a]	17.7 ± 6.4[a]	0.1 ± 0.0[a]	0.1 ± 0.0[a]	0.1 ± 0.0[a]
Ca^{2+}	2.5 ± 0.5[a]	2.6 ± 1.0[a]	3.2 ± 0.5[a]	18.3 ± 2.0[a]	32.3 ± 33.4[a]	24.6 ± 5.0[a]	0.1 ± 0.0[a]	0.04 ± 0.02[a]	0.02 ± 0.01[b]
NH_4^+	7.6 ± 2.7[a]	9.7 ± 8.5[a]	27.8 ± 21.9[a]	2.8 ± 0.3[a]	4.5 ± 5.9[a]	12.3 ± 15.2[a]	0.9 ± 0.1[a]	0.8 ± 0.1[a]	0.6 ± 0.1[b]
Cl^-	55.1 ± 39.5[a]	39.0 ± 7.4[a]	99.1 ± 36.4[a]	32.8 ± 5.4[a]	32.1 ± 11.9[a]	28.3 ± 4.3[a]	0.7 ± 0.3[a]	0.4 ± 0.3[b]	0.5 ± 0.1[ab]
PO_4^{3-}	9.7 ± 2.8[a]	14.1 ± 2.3[a]	37.4 ± 30.3[a]	19.5 ± 4.4[a]	7.5 ± 5.2[b]	2.5 ± 1.1[b]	0.2 ± 0.3[a]	0.2 ± 0.2[a]	0.01 ± 0.01[a]
SO_4^{2-}	14.7 ± 1.7[a]	23.4 ± 8.4[a]	33.1 ± 24.8[a]	18.0 ± 6.0[a]	15.0 ± 7.1[a]	4.4 ± 1.2[b]	0.2 ± 0.1[a]	0.1 ± 0.1[b]	0.2 ± 0.1[a]

Supplementary Table S9: Starch content (measured as glucose equivalent) of leaves and nectaries of different *Nicotiana* species. The four tables present the starch data from *N. africana* (A), *N. tabacum* (B), *N. sylvestris* (C), and *N. otophora* (D). FW = fresh weight

(A) *Nicotiana africana*

Treatment	Starch content in leaves [µmol/g FW]	Starch content in nectaries [µmol/g FW]
control	1.1	2.5
control	1.2	4.1
control	1.6	3.5
control	1.8	3.7
mild	2.0	3.0
mild	1.2	3.7
mild	1.4	4.5
mild	1.0	2.1
severe	0.6	4.8
severe	0.6	2.2
severe	0.7	5.8
severe	0.4	6.4

(B) *Nicotiana tabacum*

Treatment	Starch content in leaves [µmol/g FW]	Starch content in nectaries [µmol/g FW]
control	1.4	1.8
control	1.5	4.0
control	1.4	3.8
control	1.2	2.8
mild	1.3	1.2
mild	1.9	2.7
mild	1.0	6.7
mild	1.4	7.4
severe	1.8	8.1
severe	1.1	9.0
severe	1.2	8.4
severe	1.2	8.0

(C) ***Nicotiana sylvestris***

Treatment	Starch content in leaves [μmol/g FW]	Starch content in nectaries [μmol/g FW]
control	1.8	8.8
control	1.0	6.6
control	1.9	5.3
control	1.0	5.4
mild	1.1	5.8
mild	1.2	4.2
mild	1.0	6.9
mild	1.0	7.8
severe	1.2	7.4
severe	1.7	6.8
severe	1.4	5.2
severe	1.0	5.1

(D) ***Nicotiana otophora***

Treatment	Starch content in leaves [μmol/g FW]	Starch content in nectaries [μmol/g FW]
control	3.7	8.2
control	3.5	9.6
control	3.2	7.1
control	3.4	11.2
mild	4.6	10.4
mild	4.4	10.2
mild	4.2	10.0
mild	3.6	9.8
severe	3.8	9.9
severe	2.7	10.6
severe	3.3	9.5
severe	3.9	10.9

Supplementary Table S10: Results of the PERMANOVA and PERMDISP of the day- (*N. africana*, *N. tabacum*) and night-flowering *Nicotiana* species (*N. sylvestris*, *N. otophora*) separated to leaf data. The significance level (*) for PERMDISP is p ≤ 0.001.

	**Degrees of Freedom (df)**	**Pseudo-F (F)**	**R^2**	**PERMANOVA *p*-value**	**PERMDISP *p*-value**
Nicotiana africana					
Drought treatment	2	22.88	0.84	0.001***	0.655
Residuals	9		0.16		
Total	11		1.00		
Nicotiana tabacum					
Drought treatment	2	3.20	0.42	0.011**	0.435
Residuals	9		0.58		
Total	11		1.00		
Nicotiana sylvestris					
Drought treatment	2	4.92	0.52	0.001***	0.052
Residuals	9		0.48		
Total	11		1.00		
Nicotiana otophora					
Drought treatment	2	13.86	0.76	0.001***	0.092
Residuals	9		0.24		
Total	11		1.00		

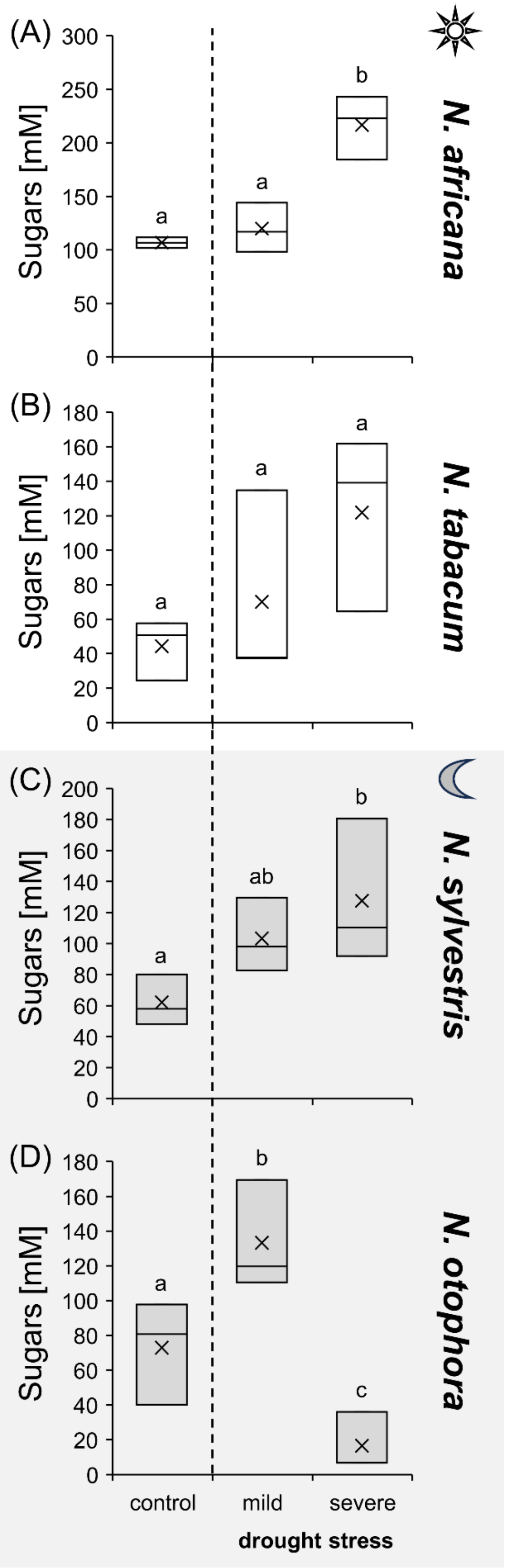

Supplementary Figure S1: Sugar concentrations in leaves of four *Nicotiana* species under different drought treatment (control, mild, severe). The *Nicotiana* species include two day-flowering (A, B) and two night-flowering species (C, D). The day-flowering species are *N. africana* (A) and *N. tabacum* (B). The night-flowering species are *N. sylvestris* (C) and *N. otophora* (D). Different letters represent significant differences in sum of sugars, respectively, between the treatments with drought (Tukey's HSD; $p < 0.05$; n=3).

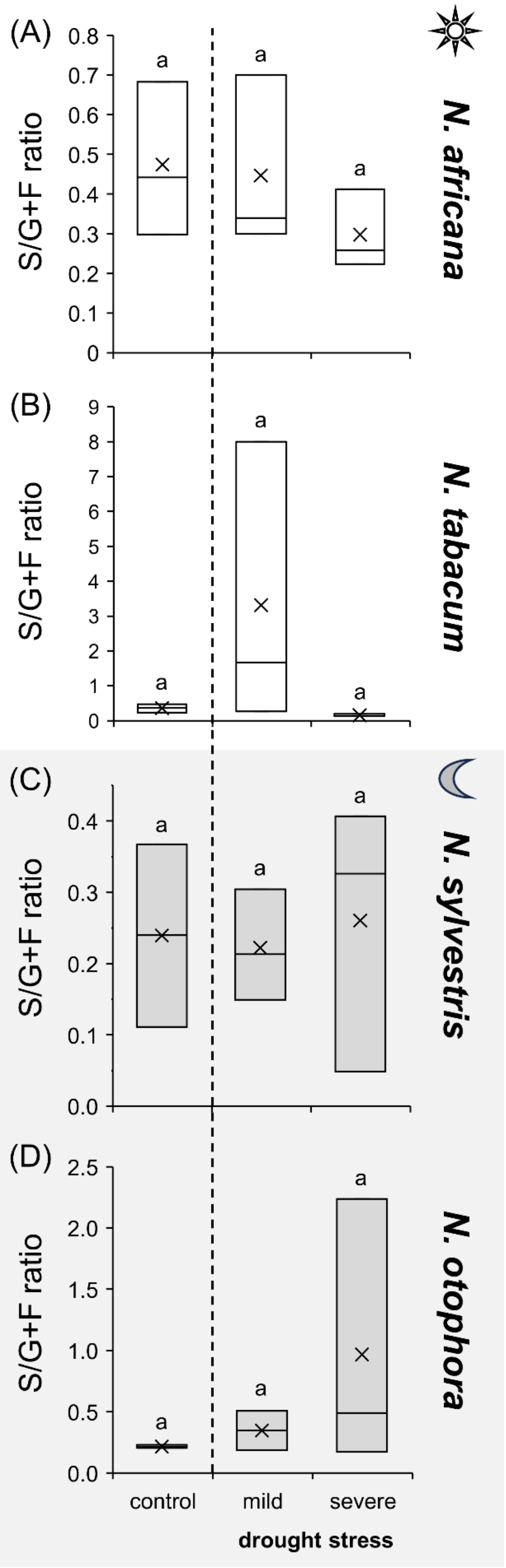

Supplementary Figure S2: Sucrose-to-hexoses ratio (ref. mM) in leaves of four *Nicotiana* species under different drought treatment (control, mild, severe). The *Nicotiana* species include two day-flowering (A, B) and two night-flowering species (C, D). The day-flowering species are *N. africana* (A) and *N. tabacum* (B). The night-flowering species are *N. sylvestris* (C) and *N. otophora* (D). Different letters represent significant differences in S/G+F ratio, respectively, between the treatments with drought (Tukey's HSD; $p < 0.05$; n=3).

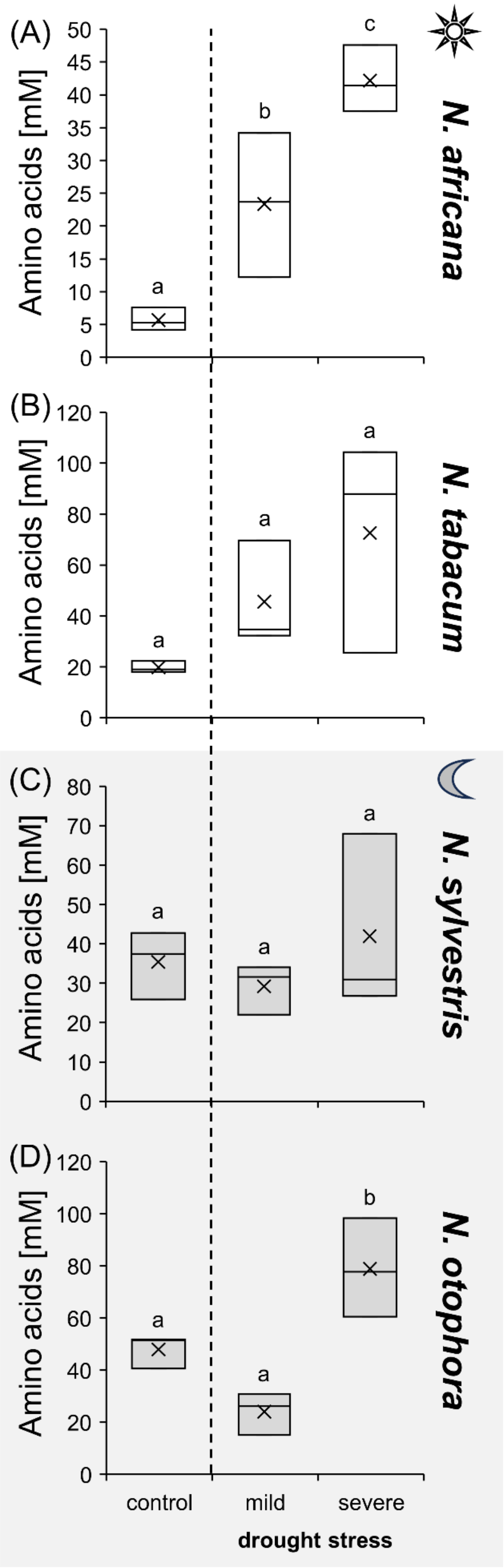

Supplementary Figure S3: Amino acid concentrations in leaves of four *Nicotiana* species under different drought treatment (control, mild, severe). The *Nicotiana* species include two day-flowering (A, B) and two night-flowering species (C, D). The day-flowering species are *N. africana* (A) and *N. tabacum* (B). The night-flowering species are *N. sylvestris* (C) and *N. otophora* (D). Different letters represent significant differences in sum of amino acids, respectively, between the treatments with drought (Tukey's HSD; $p < 0.05$; n=3).

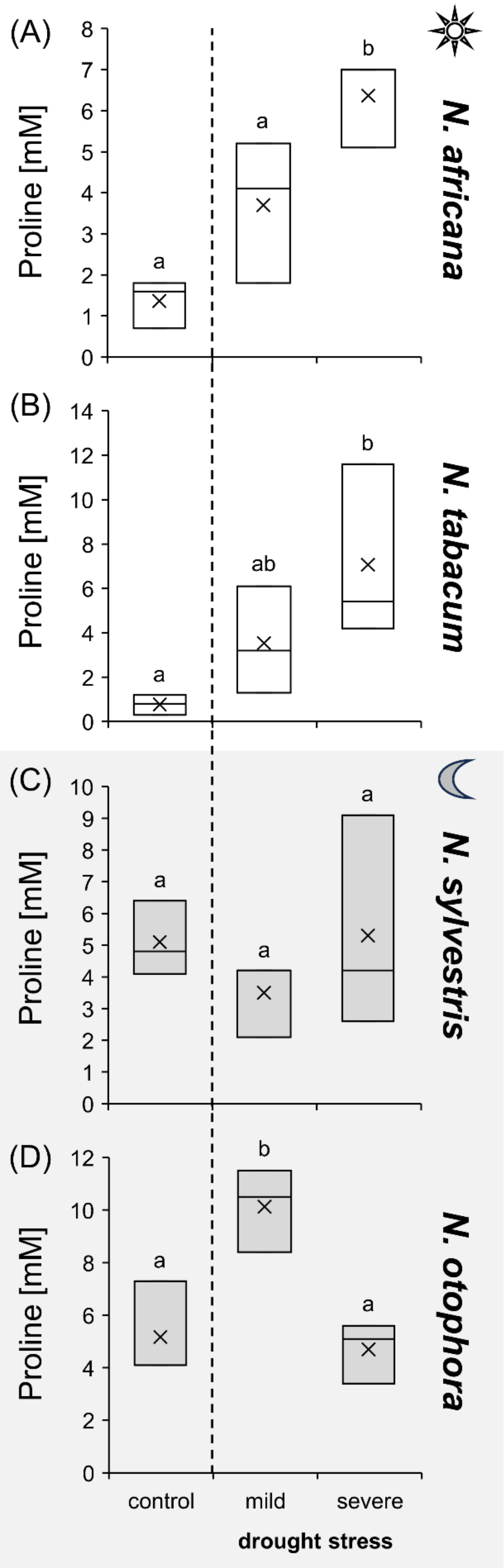

Supplementary Figure S4: Proline concentrations in leaves of four *Nicotiana* species under different drought treatment (control, mild, severe). The *Nicotiana* species include two day-flowering (A, B) and two night-flowering species (C, D). The day-flowering species are *N. africana* (A) and *N. tabacum* (B). The night-flowering species are *N. sylvestris* (C) and *N. otophora* (D). Different letters represent significant differences in proline concentration, respectively, between the treatments with drought (Tukey's HSD; $p < 0.05$; n=3).

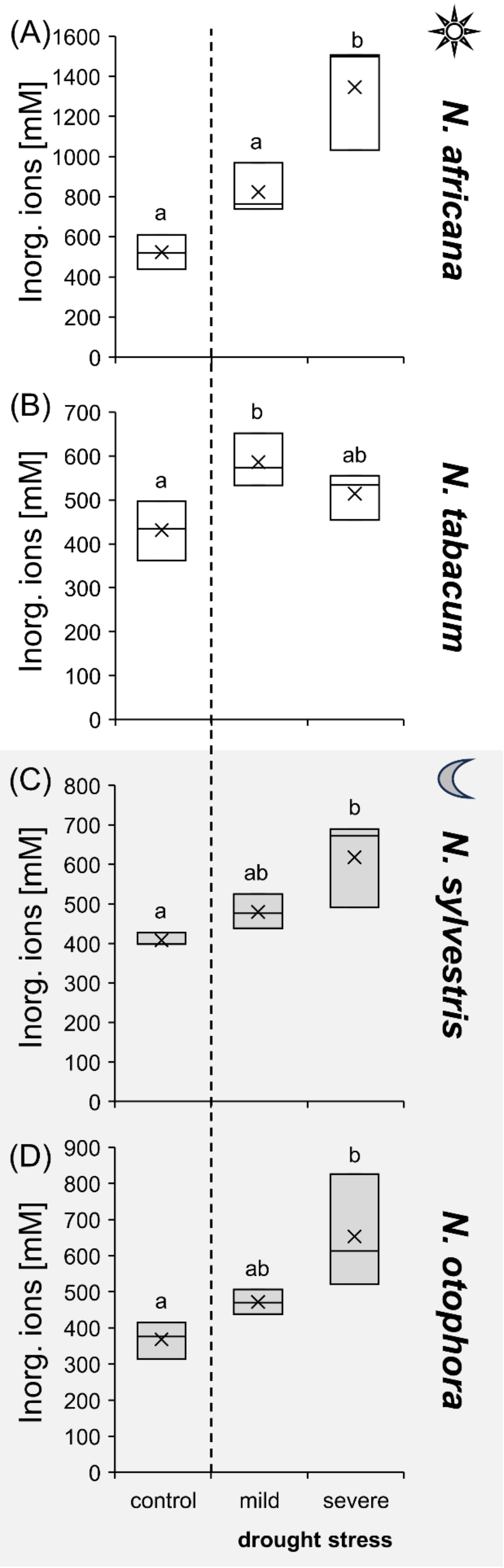

Supplementary Figure S5: Inorganic ion concentrations in leaves of four *Nicotiana* species under different drought treatment (control, mild, severe). The *Nicotiana* species include two day-flowering (A, B) and two night-flowering species (C, D). The day-flowering species are *N. africana* (A) and *N. tabacum* (B). The night-flowering species are *N. sylvestris* (C) and *N. otophora* (D). Different letters represent significant differences in sum of inorganic ions, respectively, between the treatments with drought (Tukey's HSD; $p < 0.05$; n=3).

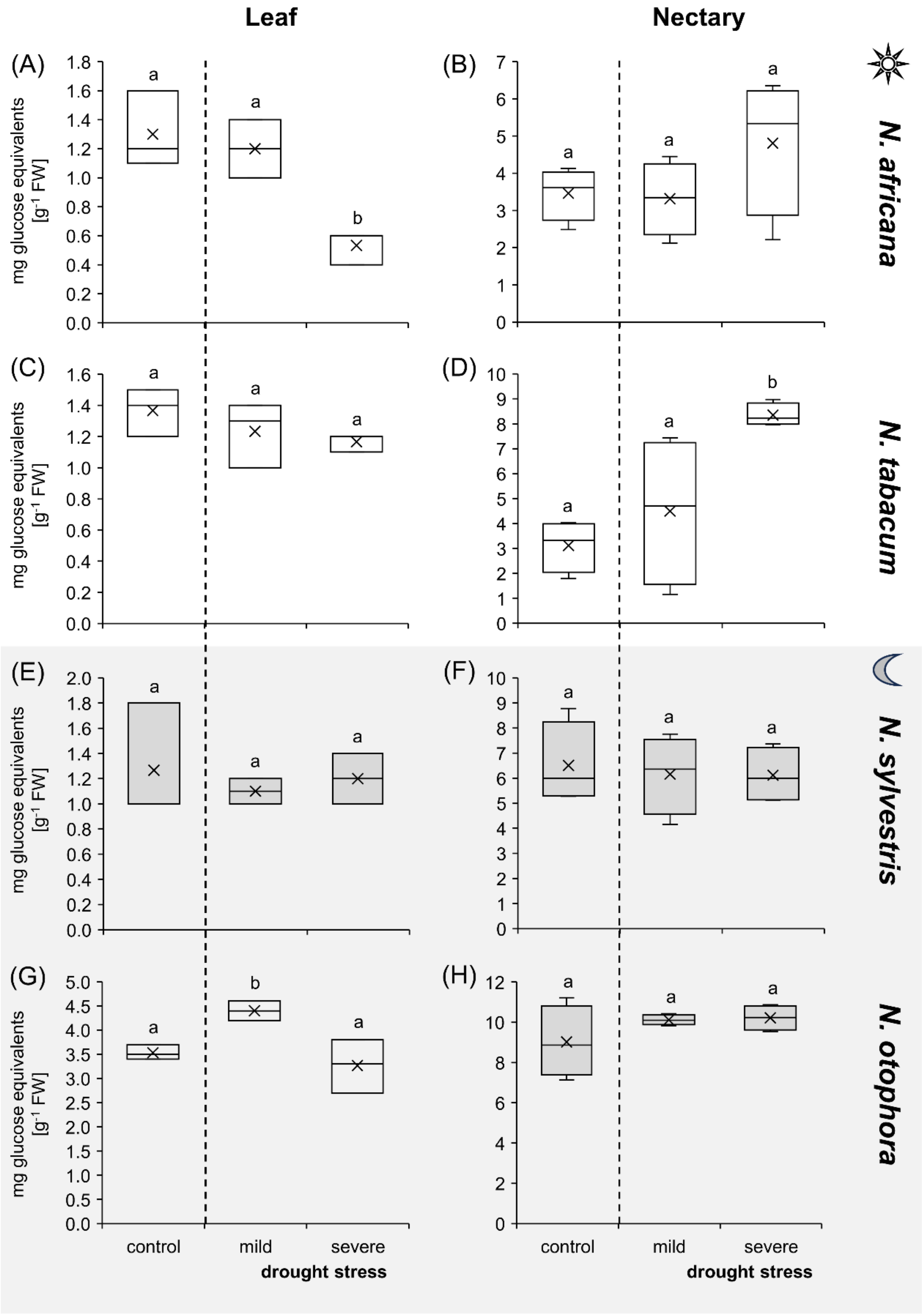

Supplementary Figure S6: Starch content measured as mg glucose equivalents g^{-1} FW in leaves (A, C, E, G) and nectaries (B, D, F, H) of four *Nicotiana* species under different drought treatment (control, mild, severe). The *Nicotiana* species include two day-flowering (A-D) and two night-flowering species (E-H). The day-flowering species are *N. africana* (A, B) and *N. tabacum* (C, D). The night-flowering species are *N. sylvestris* (E, F) and *N. otophora* (G, H). Different letters represent significant differences in starch, respectively, between the treatments with drought (Tukey's HSD; $p < 0.05$; leaf n=3; nectaries n=4). FW = fresh weight

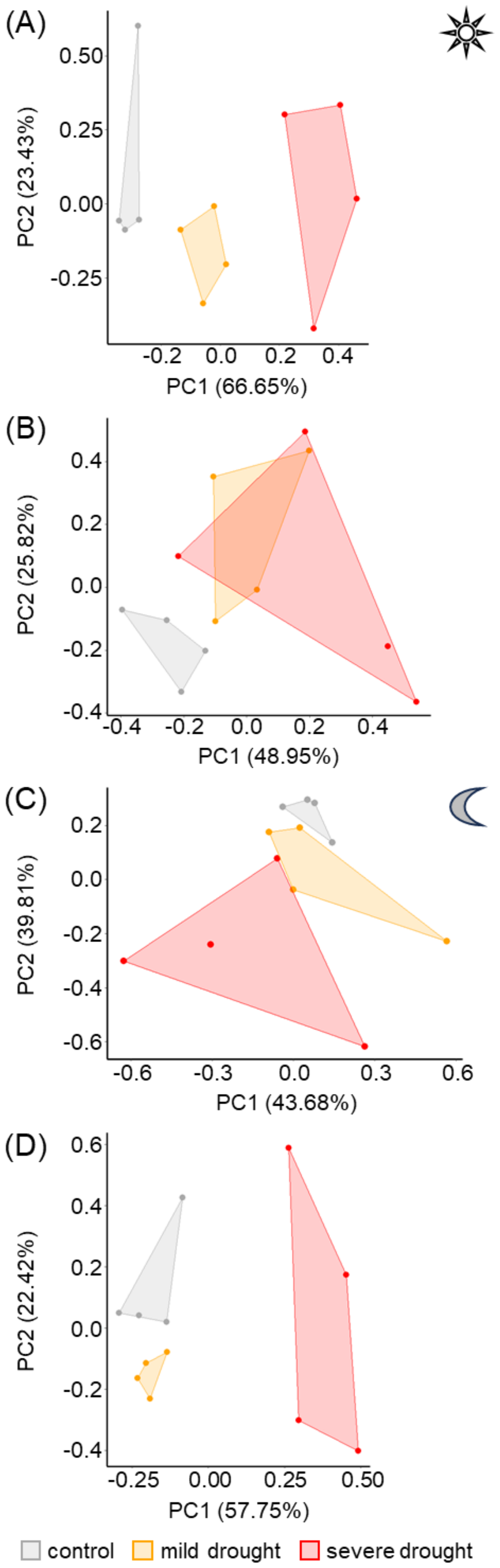

Supplementary Figure S7: Scatterplots of Principal Component Analysis (PCA) of leaf data from four Nicotiana species of different drought treatments. Sum of sugar, amino acid and inorganic ion data in nectaries were used for analysis by PCA (n=4 per treatment). The PCAs are divided into day- (A & B) and night-flowering (C & D) species. The four PCAs use the leaf data from *N. africana* (A), *N. tabacum* (B), *N. sylvestris* (C), and *N. otophora* (D).
